# Elucidation of the Landscape of Alternatively Spliced Genes and Features in the Dorsal Striatum of Aggressive/Aggression-Deprived Mice in the Model of Chronic Social Conflicts

**DOI:** 10.3390/genes14030599

**Published:** 2023-02-27

**Authors:** Vladimir Babenko, Olga Redina, Dmitry Smagin, Irina Kovalenko, Anna Galyamina, Natalia Kudryavtseva

**Affiliations:** 1 Institute of Cytology and Genetics, Siberian Branch, Russian Academy of Sciences, 630090 Novosibirsk, Russia; 2 Pavlov Institute of Physiology, Russian Academy of Sciences, 199034 St. Petersburg, Russia

**Keywords:** chronic social conflicts model, aggression, dorsal striatum, cAMP cascade, alternative splicing

## Abstract

Both aggressive and aggression-deprived (AD) individuals represent pathological cases extensively studied in psychiatry and substance abuse disciplines. We employed the animal model of chronic social conflicts curated in our laboratory for over 30 years. In the study, we pursued the task of evaluation of the key events in the dorsal striatum transcriptomes of aggression-experienced mice and AD species, as compared with the controls, using RNA-seq profiling. We evaluated the alternative splicing-mediated transcriptome dynamics based on the RNA-seq data. We confined our attention to the exon skipping (ES) events as the major AS type for animals. We report the concurrent posttranscriptional and posttranslational regulation of the ES events observed in the phosphorylation cycles (in phosphoproteins and their targets) in the neuron-specific genes of the striatum. Strikingly, we found that major neurospecific splicing factors (*Nova1*, *Ptbp1*, *2*, *Mbnl1*, *2*, and *Sam68*) related to the alternative splicing regulation of cAMP genes (*Darpp-32*, *Grin1*, *Ptpn5*, *Ppp3ca*, *Pde10a*, *Prkaca*, *Psd95*, and *Adora1*) are upregulated specifically in aggressive individuals as compared with the controls and specifically AD animals, assuming intense switching between isoforms in the cAMP-mediated (de)phosphorylation signaling cascade. We found that the coding alternative splicing events were mostly attributed to synaptic plasticity and neural development-related proteins, while the nonsense-mediated decay-associated splicing events are mostly attributed to the mRNA processing of genes, including the spliceosome and splicing factors. In addition, considering the gene families, the transporter (Slc) gene family manifested most of the ES events. We found out that the major molecular systems employing AS for their plasticity are the ‘spliceosome’, ‘chromatin rearrangement complex’, ‘synapse’, and ‘neural development/axonogenesis’ GO categories. Finally, we state that approximately 35% of the exon skipping variants in gene coding regions manifest the noncoding variants subject to nonsense-mediated decay, employed as a homeostasis-mediated expression regulation layer and often associated with the corresponding gene expression alteration.

## 1. Introduction

The studies on the neurological mechanisms of aggression along with the depression state attract specific attention in psychiatry. Aggression is coupled with an addiction-like state associated with a withdrawal (deprivation) phenomenon [1,2,3,4].

Alternative splicing (AS) has been proven to be the most abundant in the brain [5], which features the largest arsenal of neurospecific splicing factors that influence thousands of AS exons in neurospecific genes, accommodating or abrogating their inclusion into the processed transcript [5]. Consequently, many AS-mediated diseases associated with nervous tissues are reported, including spinal muscular atrophy (SMA), resulting from *Smn2* missplicing [5]; autism spectral disorder (ASD), determined by a low expression of *nSR100* (*Srrm4*) splicing factor; and many others [5].

The dorsal striatum (DS) controls the motor activity and stereotypical behaviors. It is also involved in cognitive, reward, and social hierarchy maintenance and learning [6]. In particular, the DS is proven to supervise the consolidation of the new response to learning process [6]. Hence, this brain region is inherently involved in addictive, depressive, and aggressive behaviors [7,8] when regularly exposed to a stressful environment or substance abuse.

The major body of the DS comprises GABAergic medium spiny neurons (MSNs), acting synchronously in the response to phasic dopamine [9]. In particular, we have earlier used RNA-seq transcriptome data sampled from the DS of mice in experiments with chronic social conflicts and showed distinct coordinately expressed gene cluster profiles corresponding to D1- and D2-MSNs in different phases [4,10]. The major mechanism of MSN cellular signal processing is the cAMP-mediated (de)phosphorylation signaling cascades incorporating multiple phosphoproteins [7,11,12]. We may name the range of alternatively spliced genes, such as *Darpp-32*, *Grin1*, *Ptpn5*, *Ppp3ca*, *Pde10a*, *Prkaca*, *Adora1*, etc., maintaining cAMP cascade pathways in the DS, previously reported [10] (Figure 5 therein).

Notably, phosphorylation signaling and AS are interconnected, in particular, in the cAMP-related cascades [13]. Namely, the activity of both the core spliceosomal components and accessory splicing factors is modulated by their reversible phosphorylation [14]. In turn, the subunits of protein kinases and phosphatases themselves undergo extensive AS at a transcript processing level followed by (de)phosphorylation markup, rendering the highly tuned interaction of both processes in phosphorylation-mediated signal transduction cascades [15].

Another factor affecting AS at the transcriptional level is the chromatin environment of a gene. Both the histone acetylation/methylation marks and 3D chromatin architecture of the gene region affect the splicing outcome, in particular, in neurons [16]. For example, one of the key neuronal genes, *Cacna1b*, mediates its exon inclusion by a *ctcf* factor bound in its vicinity [17]. Note that *ctcf* mediates chromatin conformation and thus changes the splicing pattern [18]. The *PolII* transcription rate is greatly affected by the chromatin environment following the *PolII*-coupled splicing decisions [19].

AS in mammals is mostly manifested by the genes of neurons along with muscle-specific genes, but to a lesser extent [20]. A range of AS events manifested by exon skipping/insertion leads to the premature emergence of a stop codon [21] and is subject to nonsense-mediated decay (NMD) [22], serving as an additional means of the prompt expression abrogation upon the local compartment dynamics of protein turnover. It is employed in the maintenance of homeostasis of multicomponent complex subunits, such as the ribosome, spliceosome, and chromatin remodeling machinery specifically in the brain cells, where NMD regulatory splicing events were prevalent [23].

Using the same data on the social conflict model of repeated aggression-exposed mice with positive (winning) experience (A20), consequent aggression deprived (AD) mice, and control groups (see Methods), we recently assessed the differentially expressed genes (DEGs [4]) emphasizing the expression profile specifics of each group. The unexpected straight outcome of the study shows that while the A20 group differed from the control by over 1000 DEGs, the AD group (14 days of deprivation span after A20 group protocol) expression profile was quite similar to that of the control (62 DEGs [4]). The control vs. AD group DEGs manifested an expression network of the circadian rhythm genes that significantly deviated from the control in both the A20 and AD groups.

The ultimate goal of this work was to assess the differentially alternatively spliced (DAS) genes in the involved groups and to gain an insight into the overall AS-mediated transcriptional diversity landscape features within the DS with outlining of the major AS-enriched gene networks.

## 2. Materials and Methods

### 2.1. Animals

Adult male C57BL/6 mice were obtained from the Animal Breeding Facility with the Institute of Cytology and Genetics, Siberian Branch, Russian Academy of Sciences (Novosibirsk, Russia). Animals were housed under standard conditions (12/12 h light/dark regime starting at 8:00 a.m. with food in pellets and water ad libitum). Mice were weaned at 3 weeks of age and housed in groups of 8–10 in standard plastic cages (36 × 23 × 12 cm). Experiments were performed with 10–12-week-old animals weighing 26–28 g. All procedures were in compliance with the European Community Council Directive 210/63/EU of 22 September 2010. The study was approved by the Scientific Council no. 9 of the Institute of Cytology and Genetics of 24 March 2010, no. 613 (Novosibirsk, Russia).

### 2.2. Experimental Procedures

#### 2.2.1. Modeling Repeated Aggression in Male Mice

Repeated negative and positive social experiences in male mice were induced by daily agonistic interactions with the use of the sensory contact model, which later was renamed as the “model of chronic social conflicts” and is comprehensively described in [24,25]. Each pair of male mice were placed in a cage (28 × 14 × 10 cm) bisected with a perforated transparent partition allowing the animals to hear, see, and smell each other but preventing physical contact. The animals were left undisturbed for 2 days to adapt to the new housing conditions and sensory acquaintance before they were exposed to agonistic interactions. Every afternoon (2:00–5:00 p.m. local time), the cage cover was replaced with a transparent one, and 5 min later (the time it takes for a mouse to start reacting to a partner in the neighboring compartment), the partition was removed for 10 min to encourage agonistic interactions. The superiority of one of the mice was promptly established within two or three confrontations with the same opponent. The superior mouse (winner) would be attacking, chasing, and biting another, who would display only a defensive behavior (withdrawal, sideways postures, upright postures, freezing, or lying on the back). As a rule, aggressive interactions between males were discontinued by lowering the partition if the strong attacks lasted 3 min (in some cases, less) to prevent damage to the defeated mice. Each defeated mouse (loser) was exposed to the same winner for 3 days, while afterwards, each loser was placed once a day after the agonistic interactions in an unfamiliar cage with a strange winner behind the partition. Each winning mouse (aggressive mice, winner) remained in its original cage. This procedure was performed for 20 days (once a day) and yielded an equal number of losers and winners.

In each experiment, we tracked the behavior of all males, making videos of the behavior during agonistic interactions, which allowed us to identify the most aggressive mice that demonstrate the greatest daily number and duration of attacks, hyperactivity, the number of stereotypes, etc. The winners with the most eminent aggressive phenotypes (long lasting daily expressed aggression towards any losers) were selected for the transcriptome analysis. The winners after a period of 14 days deprivation demonstrated increased aggression as compared with the aggression level before deprivation [1,2].

Notably, a chronic aggression accompanied by a positive reward (win) inherently manifests an addiction state under a positive fighting experience in mice [1,2,3]. All signs of addiction development behavior, similar to drug users according to Robinson and Berridge [26], feature in the mice with repeated experience of aggression supported by wins in daily agonistic interactions [3,26]. In particular, we observed the activation of brain dopaminergic and opioidergic systems via the development of tolerance or sensitization to the antagonists of dopaminergic and opioidergic receptors or agonists after chronic aggression experience [3]. Similar to the withdrawal effect of substance abusers, the experienced winners demonstrated increased aggression (stress) after the period of fighting deprivation [3].

**Figure 1:**
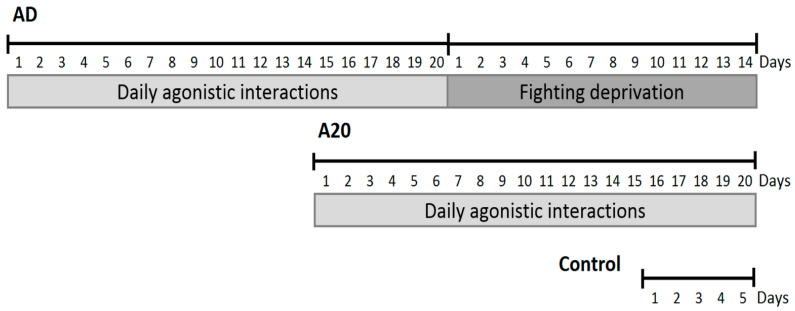
Timescale of the experiment. A20 are the mice with 20-day repeated aggression accompanied by wins in daily agonistic interactions; AD, A20 aggression-deprived mice after 14 days of fighting deprivation; control, mice without consecutive agonistic interactions.

Three groups of animals (males) comprising six mice each were analyzed in this experiment (Figure 1): (1) controls, the mice without a consecutive experience of agonistic interactions; (2) winners, groups of repeatedly 20-day aggressive mice (A20); and (3) aggression-deprived mice (AD), converted from winners after a period of fighting deprivation during 14 days in secluded cages. The winners 24 h after the last agonistic interaction, control animals, and AD were simultaneously euthanized by decapitation. The brain DS regions were dissected by the same experimenter according to the map in the Allen Mouse Brain Atlas [27] (assessed on 24 April 2005). All samples were deposited in RNA later solution (Life Technologies, Carlsbad, CA, USA) and stored at –70 °C prior to sequencing. No technical replicates were performed.

#### 2.2.2. RNA-Seq Data Collection and Processing

The collected brain samples were delivered to the JSC Genoanalytica (www.genoanalytica.ru (accessed on 10 June 2020), Moscow, Russia) for routine RNA-seq. The mRNA was extracted using a Dynabeads mRNA Purification Kit (Ambion, Thermo Fisher Scientific, Waltham, MA, USA). The cDNA libraries were created using the NEBNext mRNA Library PrepReagent Set for Illumina (New England Biolabs, Ipswich, MA, USA) according to the manufacturer’s protocol. The Illumina HiSeq 2500 System was used for sequencing with the help of single (non-paired end) reads with a length of 50 bp. The target coverage was set to 30 million reads per sample on the average.

The raw reads from RNA-seq experiments were trimmed for quality (phred ≥ 20) and length (bp ≥ 32) using Trimmomatic v. 3.2.2 [28], including Illumina adapters. The reads were then aligned against the GRCm38.p3 reference genome using the STAR aligner [29]. The statistics of sample mapping is available in Table S1.

#### 2.2.3. Analysis of Alternative Splicing

**Figure 2:**
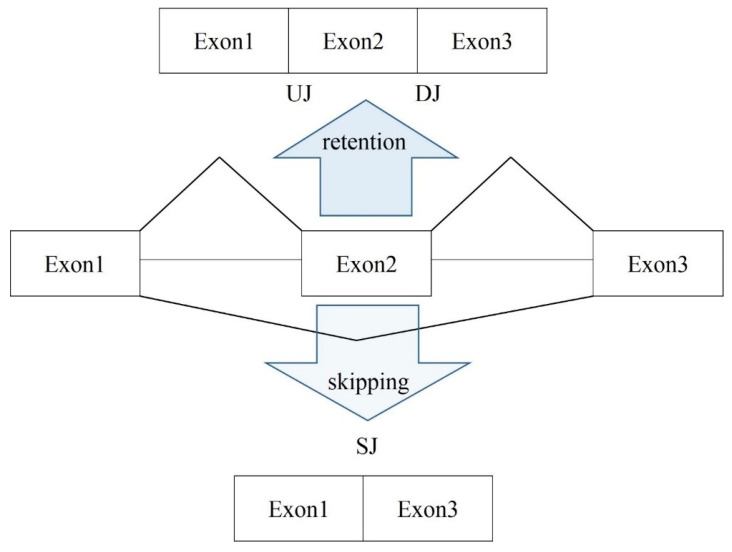
Scheme of exon skipping identification. Three consecutive exons are employed. The reads overlapping exon junctions are selected. UJ is upstream (exon) junction; DJ, downstream (exon) junction; and SJ, skipping (exon) junction. The coverage rate of the corresponding junctions allow for the inference of the major isoform. Lack of SJ reads implies that no ES event is observed at the site.

While there are many reported types of AS, we focused on the ES events since this is the prevalent AS type in animals [30,31]. The detection algorithm for ES events was first introduced on expressed sequence tags (EST) data [32], and later applied to RNA-seq. It employs three consequent exon RNA segments and analysis of the junction overlapping reads (Figure 2).

Alternatively spliced exons in each sample were assessed with rMATs software [33]. We used the parameters adjusted for 50 bp reads: dataType = single (non-paired end tags); read Length = 49; anchor Length = 8; and junction Length = 82; Inc Form Len = 2 × (j − r + 1) = 2 × (82 − 49 + 1) = 68; and Skip Form Length = 34.

The joint likelihood function of the statistical model in rMATs is a combination of the binomial distribution modeling the relationship of the exon inclusion reads, exon skipping reads, and the exon inclusion level in each individual replicate and the normal distribution modeling the variation of the replicate exon inclusion levels within the sample group. Thus, the joint likelihood function is composed of two components.

The exon inclusion distribution model implies the introduction of a key exon inclusion rate statistic *ψ_I_* by the equation accounting for the observed inclusion (*I*) and skipping (*S*) the read numbers and the lengths of *l_I_* and *l_S_* of the corresponding effective reads:

$$\psi_{I}=\frac{I/l_{I}}{\frac{I}{l_{I}}+\frac{S}{l_{s}}}:$$

Assuming a binomial distribution for exon inclusion rate, we obtain:

(1) $$F{\left(\psi \right)}^{}=\sum_{k=0}^{I+S}\left(\begin{array}{c} I+S\\ k \end{array}\right)f{\left(\psi \right)}^{k}{\left(1-f\left(\psi \right)\right)}^{I+S-k},$$

where $f\left(\psi_{I}\right)$ is the normalized inclusion rate *ψ_I_*: $f\left(\psi_{I}\right)=\frac{l_{I}\psi_{I}}{l_{I}\psi_{I}+l_{s}\left(1-\psi_{I}\right)}$.

The (non-paired) replicates within each group are used to assess the exon inclusion rate variance within the group, assuming a normal distribution [33]:

(2) $$logit\left(\psi_{I}\right)\sim Normal(\mu =logit\left(\psi_{I}\right),\;\sigma^{2})$$

The log likelihood test checks if the probability of the between-group deviation of exon inclusion ratios is less than the predefined threshold *e*: $|\psi_{I1}-\psi_{I2}|<e,$ given the within-group variation (2).

#### 2.2.4. In-House Script for the Checkup of the Coding Potential of Exon Skipping Events

We created a Perl script pipeline, which downloads and merges three consecutive exon sequences with a targeted ES-associated middle exon (Figure 2) in the corresponding orientation and translates it in three possible reading frames in order to determine the coding ORFs. The translated polypeptides are then compared to the protein database elucidating the target annotated variant. The coding potentials of both included and skipped isoforms were assessed in this way.

#### 2.2.5. Statistical Analysis

Principal component analysis (PCA) was employed using the XLStat statistical package (www.xlstat.com; accessed on 1 May 2022). The Pearson product moment correlation matrix for gene expression in samples was used as the input data for PCA. Volcano plot utility was also created with XLStat software.

#### 2.2.6. Workflow Schema of the Analysis

**Figure 3:**
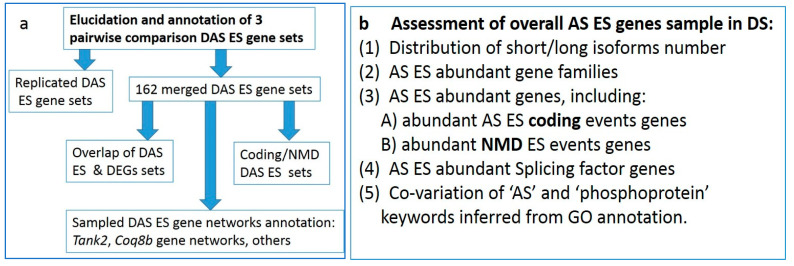
Workflow scheme for data analysis: (**a**) DAS genes analysis; (**b**) whole genome AS capable gene analysis.

LA logical scheme of analysis consists of two subsections: analysis of DAS ES genes (Figure 3a) and the genome wide pool of splicing manifested genes (Figure 3b). We decided to make it explicit for better orientation in the workflow logics throughout the manuscript.

## 3. Results

After identifying the AS exon skipping (ES) events in RNA-seq data with rMATs software [33], we revealed that the assayed DS samples contained somewhat equal number of the total ES events, which was around 7000 (Table 1). Along with the ES events, there were approximately 3700 alternative 5′/3′ exon boundaries and 420–450 mutually exclusive ES events (MXEs), depending on the sample. Note that the alteration of 5′/3′ exon boundaries was the major source of the poison exons (with a shifted coding frame) that emerged in our data. The vast majority of them was quite rare (one to three ES events per sample) and were filtered out in the further analysis if the total ES instances (number of confirmed overlapping junction reads; Figure 2) were less than 10 across the set of total samples.

All identified ES events were checked up against the annotated ones in the *knownAlt* table of mm10 genome database (https://hgdownload.soe.ucsc.edu/goldenPath/mm10/database/knownAlt.txt.gz (assessed on 15 November 2021). This revealed an excellent consistency of the annotated exons: only 14–17 exons of each comparison (comprising in total approximately 7000; Table 1) were not annotated as the ‘*cassetteExon*’ class in ‘*knownAlt*’ table (genome.ucsc.edu) for each pairwise comparison.

We assigned the statistically significant DAS ES events with an FDR < 0.11 threshold in three pairwise comparisons of the groups by means of rMATs software (Table S2a). More than a third of DAS ES events proved to be outside of the coding part (5′/3′UTR, Table S2a). Further on, we separately assessed both the overall DAS events (see the statistics in Table 1, first column, and the full info on DAS genes in Table S2) and the ones that resided in CDS only (Table 1, third column), and annotated by the corresponding ENSEMBL protein IDs (Table S3a).

### 3.1. Annotation of DAS ES Genes

We then proceeded with the GO annotation in each group. Table S2b–d contains three corresponding GO annotations for three pairwise comparisons.

**Figure 4:**
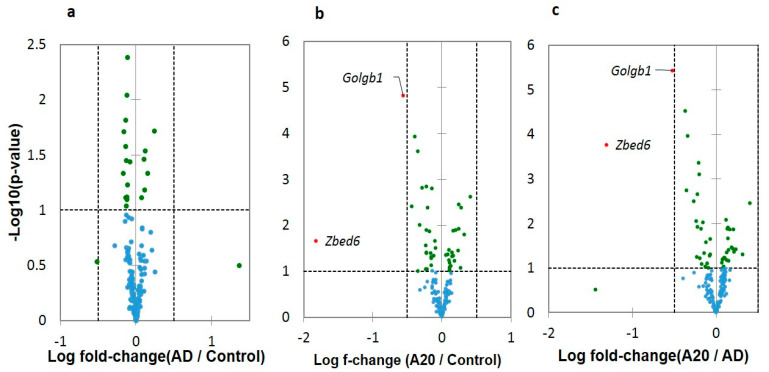
Volcano plots of the differential expression of DAS ES genes (Table S2a) created by Xlstat software (XLStat.com). (**a**) DEGs Log fold change in AD vs. control groups comparison; (**b**) DEGs Log fold change in AD vs. control groups comparison; (**c**) DEGs Log fold change in A20 vs. AD groups comparison. *Golgb1* gene is Golgin b1 Golgi autoantigen and *Zbed6* is Zinc Finger BED Domain-Containing Protein 6.

We checked for the differential expression of three DAS ES genes by pairwise comparisons using Volcano plots (Figure 4). As is evident, the vast majority of DAS ES genes do not essentially differ in a log fold change, while a distinct and significant isoform ratio is observed (Table S2a). Two DAS DEGs shown in Figure 4 relate to the overall metabolism intensity. *Zbed6* DEG is involved in the mRNA export from nucleus (GO: 0006406), and *Golgb1* in Golgi maintenance, including protein transport (GO: CL:21133). Both genes co-vary (*R* = 0.745; df = 17; *p* < 0.001) and their downturn specifically in A20 may reflect metabolism alteration [4] (Figure 10 therein). *Zbed6* further proved to be a single exon gene located within the intron of *Zc3h11a*, thus was not suitable for further analysis as a DAS gene.

### 3.2. The 163 Merged DAS Genes

#### 3.2.1. GO Annotation

**Figure 5:**
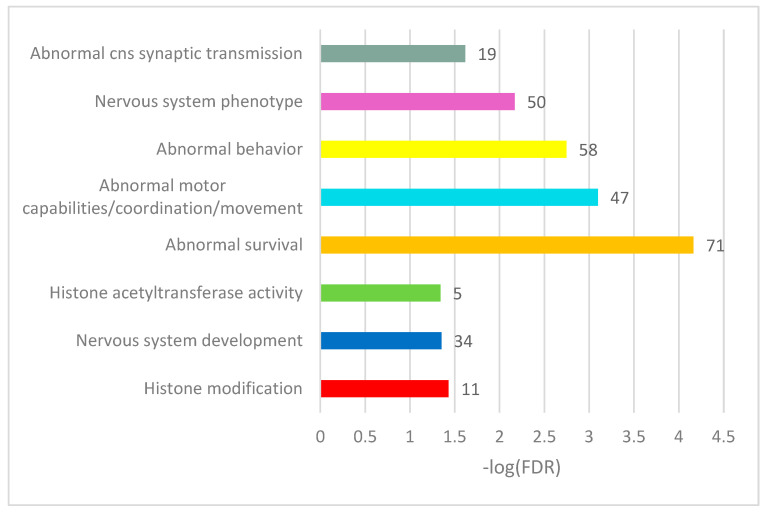
Selected GO annotation terms of 163 merged DAS ES genes (see full GO annotation in Table S2f). The number of the observed DAS ES genes is shown as bar labels.

We performed a GO annotation for the DAS genes in each comparison (Figure 4). While separate pairwise GO annotations yielded no essential results (Tables S2b–d), we compiled a list of merged DAS genes (163 unique entries) from all comparisons and annotated it (Table S2e). Herein, we report on several selected GO categories shown in Figure 5 (see full annotation in Table S2f).

Overall, we observed the consistent DAS ES events enriched in chromatin (histone) rearrangement GO categories (Tables S2a and S3b,c). Note that 71 of the 163 DAS ES genes were annotated as an enrichment with ‘abnormal survival’ genes (Figure 5), meaning ‘deviation from the expected viability or life span of an organism’ (MP:0002206; Monarch nomenclature; [34]), implying the genes are essential for an organism’s maintenance.

#### 3.2.2. Distribution of A20-Specific DAS ES Genes

**Figure 6:**
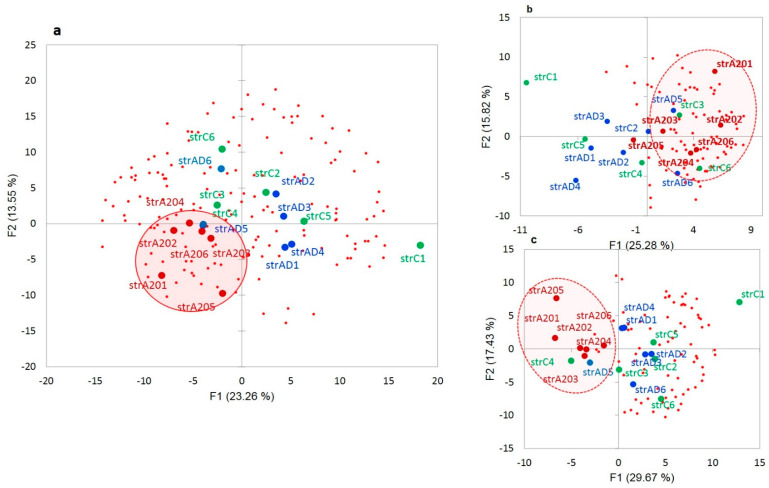
(**a**). PCA plot of the distribution of 163 merged DAS ES genes (Table S2e) based on their expression profiles. Red small dots denote the DAS genes; big red dots: AD20 group; blue dots: AD; and green dots: control. (**b**). Subset (from (**a**) genes) of 81 fold elevated A20-specific genes (Table S2g). (**c**). Subset (from (**a**) genes) 76 fold A20 negative genes (Table S2i). See GO annotation clusters (**b**,**c**) in Table S2g,j, respectively. Clustering of the A20 group is systematically observed in each plot (encircled).

We plotted the PCA distribution of DAS genes across the samples based on their expression profiles (Figure 3a). Consistent with our earlier work on the same data [4], the A20 group manifested a distinct clustering through the landscape of the gradient gene expression (Figure 6a). We further performed agglomerative hierarchical clustering (AHC) of 163 DAS ES genes, leading to two major clusters of A20 enhanced genes (81 entries; Figure 6b and Table S2g) and A20 downregulated genes (76 entries; Figure 6c and Table S2j).

**Figure 7:**
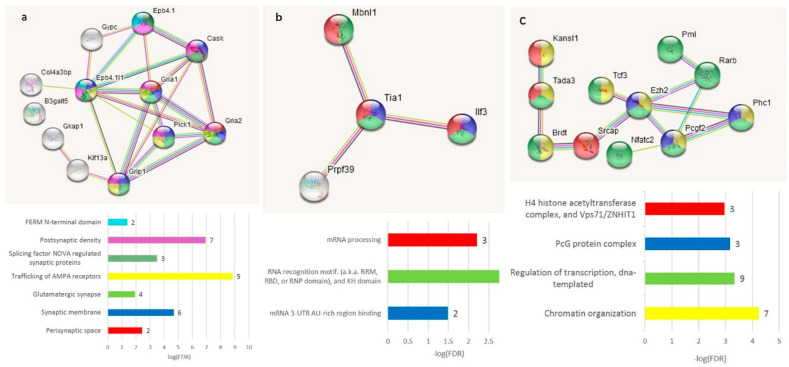
Three expanded connected neighborhoods of the DAS ES genes elevated in A20 subjects (Figure 6b) signifies (**a**) enrichment with synaptic DAS ES genes; (**b**) mRNA processing enzymes; and (**c**) chromatin rearrangement of DAS genes. Color codes correspond to the bar plot below and the number of observed DAS ES genes are shown as bar labels.

The GO annotation in the A20-associated cluster (Figure 6b) revealed the lack of nontrivial GO enrichment categories (Table S2h); correspondingly, we expanded several gene networks involved by applying the string-db.org genes set expanding option. The results are shown in Figure 7.

**Figure 8:**
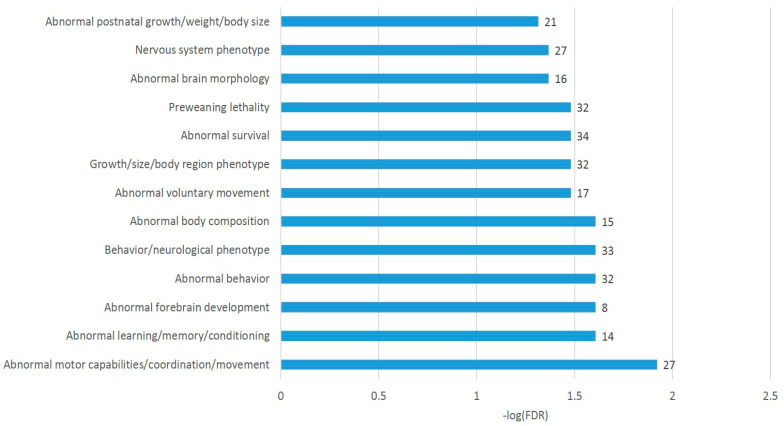
Annotation of 76 DAS ES genes attenuated in the A20 group (Figure 6c). See Table S2j for explicit GO information.

The A20 attenuated group of DAS ES genes, outlined in Figure 6c, demonstrates the enrichment of the gene networks related to the DS brain region (Figure 8). Most of the terms were retrieved from the Monarch phenotypic database [34] (Table S2j), incorporated in string-db.org.

**Figure 9:**
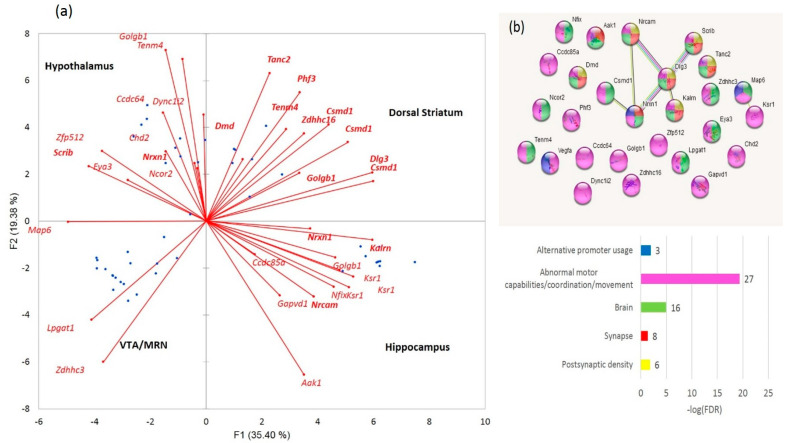
(**a**). Elucidation of eight DS-specific DAS ES genes (*Tanc2*, *Csmd1*, *Phf3*, *Dlg3*, *Tenm4*, *Zdhhc16*, *Dmd*, and *Golgb1*) from the 27 analyzed using our earlier published data on five brain regions [10]. (**b**). Explicit GO annotation of the 27 gene cluster (GO: MP:0002066; ‘Abnormal motor capabilities/coordination/movement’). VTA is the ventral tegmental area and MRN, midbrain raphe nuclei.

For gaining a more comprehensive insight into these DAS genes, we selected the DAS ES genes of the largest category in Figure 8 (GO: MP:0002066; ‘Abnormal motor capabilities/coordination/movement’; FDR < 0.012; 27 genes; Table S2j), which overlap other GO categories of gene sets in Figure 8 as well, for a detailed analysis, as shown in Figure 9. We found out that the set contains postsynaptic density genes, particularly referring to the DS (eight DAS genes; Figure 9a,b). In addition, the ES events observed within the connected network (Figure 7b, green/red shaded nodes) may imply that the splicing occurs coordinately within this network. This confirms the previous statements on essential structural synaptic density contraction in the aggressive/addictive subjects [4,6].

#### 3.2.3. DAS Genes vs. DEGs

It has been over 10 years since the links between the chromatin, PolII elongation rate, and splicing outcome were reported [19,35,36,37,38,39]. When transcription starts, it produces a pool of mRNAs that is stored unprocessed [40]. The quick homeostasis balancing challenges are addressed with the corresponding splicing processing response mediated by splicing factors (SFs), inducing the NMD cascade and abrogating effective translation of a particular gene.

Note that NMD-mediated splicing is reported to provide the feedback for attenuating/augmenting its transcription rate [41]. Thus, NMD-related events do provide association with the transcription rate along with effective translation attenuation but the overall RNA isoform entity can hinder a downturn effect at the overall transcription level.

Therefore, although there is usually no direct overlap of DAS genes and transcription-mediated DEGs because of different timescales/phases of the processes, the network of the corresponding DAS ES NMD-related genes features the changes in its overall expression rate underscored by the altered ratio of coding/noncoding isoforms with the subsequent transcription rate modulation. Based on this, we would emphasize that the time scaling of splicing and expression phases makes it particularly crucial to assess both stages in the course of transcriptome evaluation.

Another point hampering RNA-seq analysis is a burst-like pulsating mode of gene expression, which was reported recently [42,43]. This mode is widespread in genes and is often accompanied by ES NMD AS and chromatin maintenance, including the activity-inducible neuronal genes [44,45].

**Figure 10:**
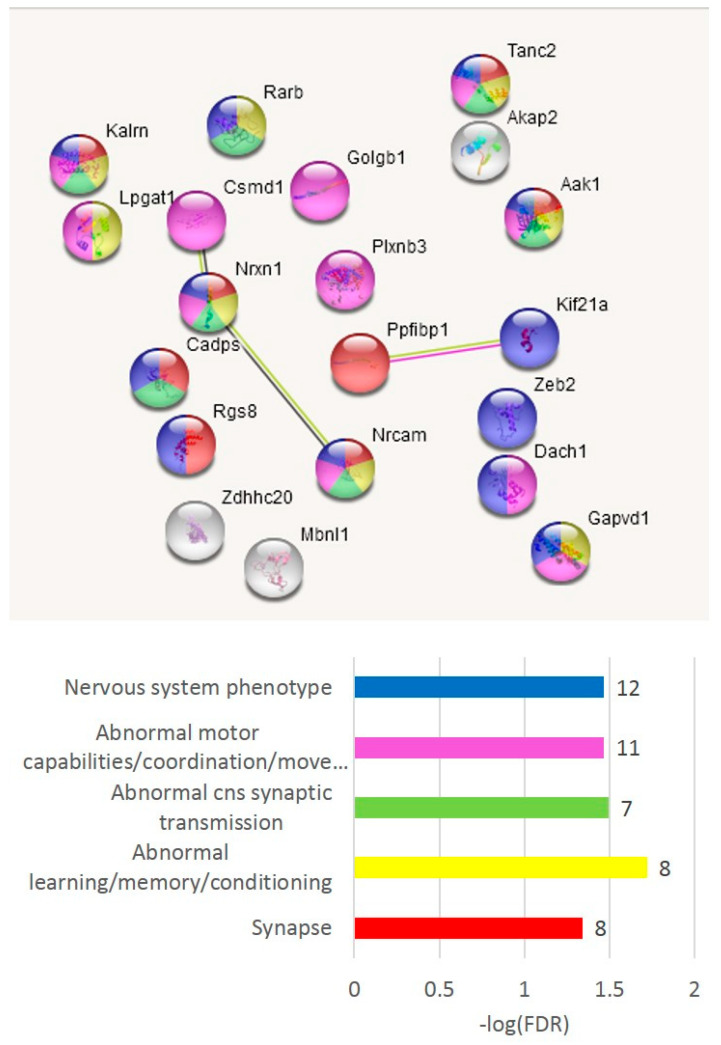
The 20 distinct DAS ES genes overlapping DEGs [4] (Table S3a,b). Color codes are shown as a bar plot with the number of DEGs/DAS genes attached as labels. We identified DAS ES *Mbnl1* SF gene, which significantly elevated its expression in aggressive subjects. The GO categories (Monarch MPO; monarchinitiative.org; embedded in string-db.org project) reflect the DS brain region phenotype features in the essential part. See the description of full GO annotation/genes in Table S3b,c.

We elucidated 20 DAS genes from the total of 163 merged (Table S2e) that overlap DEGs reported in earlier work [4] (Table S3a). Figure 10 outlines the specific GO chosen terms.

Most of the genes shown in Figure 10 are the DEGs altered particularly in the A20 group against the two other groups (Table S2k), except for *Zeb2*, which displays the DAS effect in the AD group as compared to two others (Table S3c). The 5′UTR ES target exon, not reported in public databases among multiple *Zeb2* ES isoforms as of now, overlaps the first exon of *Zeb2os* but does not fully match it in size. It was located between 5′UTR exons 2 and 3 by rMATs software.

*Zeb2* is reported to be involved in astrogliosis and accommodates the CNS recovery after an injury [46], performing also as an inhibitor of histone acetylation. It has increased expression in the AD group more than two-fold as compared with the two other groups (Table S9c).

Notably, the majority of the considered DAS genes/DEGs manifest the highest possible expression differences relative to the accuracy range (1 × 10^−5^, Table S3c), implying rather drastic expression level alterations in these genes following/preceding the ratio alteration in their significant isoforms.

For a better appreciation of the splicing alteration, we show the plots of DAS/DEG group alternative splicing genes in Table S3d. Correspondingly, this suggests that *Csmt1* has altered its major isoform in AD, while *Zeb2* maintains the major isoform with the skipped 5′UTR ES exon.

Thus, the analysis of the DAS plots (Table S3d) suggests that the majority of DAS events in the DAS/DEG group (and, in general, in the DAS gene pool as well) arise owing to the nonlinear ratio alteration of isoforms upon a change in the gene expression, which can be used as an auxiliary indicator of an expression rate impact.

#### 3.2.4. Coding/NMD DAS Events

We assessed the DAS ES events in the coding part of the genes. Table S4a comprises the corresponding protein ENSEMBL IDs for each isoform, where available. We ascertained that while 59 genes maintain both AS ES isoforms as coding (long and short ones), termed AS2 events (Table 2), there were 32 AS ES events maintaining only one of the isoforms as a coding one (NMD events).

#### 3.2.5. Replicated DAS Genes in Comparisons

To address the consistency of DAS ES events, we compiled the cross-comparison of the AS2 DAS ES common events in three pairwise comparisons, as listed in Table 3 below.

We found that 23 DAS ES events in 21 genes are replicated in at least two different comparisons (Table S4b,c), implying some consistent DAS dynamics across the whole set of the samples. We performed GO annotation in these sets.

We observed two consistent GO categories emerging across the three merged DAS sets. The replicable DAS genes in three comparisons (Table S4c) feature the chromatin remodeling enzymes (*Hdac7*, *Ezh2*, *Rarb*, *Chd2*, *Brdt*, and *Scrap*; Table S4c). *Hdac7*, the most altered DAS gene, manifests the ratio alteration of isoforms in both the A20 and AD groups (Table S4a).

The endocytosis/clathrin-coated gene network was apparently relevant in DAS genes manifestation (Table S4c).

We also utilized the string-db.org-mediated targeted gene network expansion option to increase the associated network to 31 genes (Table S4d). Four GO categories have been distinctly outlined: ‘Neural crest formation’, three genes (FDR < 0.009); ‘Chromatin organization’, seven genes (FDR < 0.016); ‘Histone h3-k27 trimethylation’, two genes (FDR < 0.034); and ‘EGFR1 signaling pathway’, four genes (FDR < 0.02; Table S4d).

Note that the chromatin-related NMD evolutionary conserved ES events for the mammalian brain were reported earlier [47]. Here, we observed both coding and NMD events across this group of genes (Table S4c).

#### 3.2.6. GO Annotation of AS2/NMD DAS ES-Related Genes

**Figure 11:**
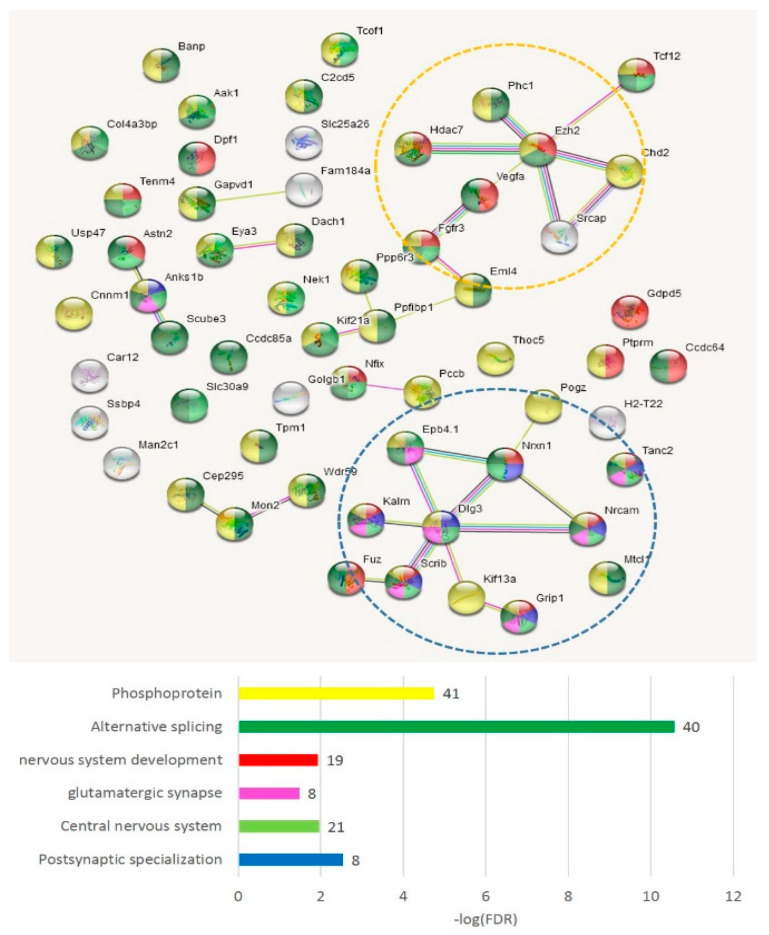
In total, 59 AS2 DAS genes (with both isoforms coding) underlie the synaptic dynamics in the DS neurons. The full GO clustering of 59 AS2 DAS genes is shown in Table S5b along with scalable Figure 11 plot (Table S5c). The cluster of glutamatergic synapse genes (blue dotted line and pink shaded genes) and the cluster of chromatin remodeling genes (yellow dotted line) are encircled. The GO enrichment statistics and color-coding scheme are shown in the bar plot (number of genes attached as bar labels).

We compiled the merged non-overlapping AS2/NMD DAS gene lists (Table S5a) and checked them against the GO annotations. We have found that the AS2 events represent mainly synaptic and neural development genes (Figure 11); 59 merged AS2 genes (Table S5a) maintain 30 edges against the expected 13 (PPI enrichment value *p* < 2.24 × 10^−5^).

As is evident from Figure 11, there are two basic machineries featuring proteins capable of differential AS: (a) chromatin remodeling in the nuclear compartment and (b) synaptic plasticity (membrane compartment) in glutamate-related and other receptors.

#### 3.2.7. NMD-Related DAS Genes

When we assessed the GO enrichment for the list of NMD-related 32-fold DAS genes (Table S5a) with the string-db.org tool, we did not observe any significant enrichment with the GO categories/networks, except for trivial ones (AS and phosphoprotein, Table S5d). We proceeded to assess several DAS ES genes by recovering the gene network using the string-db.org resource.

### 3.3. Postsynaptic Plasticity of Standalone DAS Gene-Related Receptors

While two confident gene networks were outlined with significant enrichment (Figure 8), we undertook an effort to elaborate on other single DAS genes from Figure 8 using string-db.org for recovering the context.

#### 3.3.1. Tanc2 Splicing Specifics

We observed the *Tanc2* ex4 skipped aberrant isoform with consistent emergence in all groups. It contains 26 exons (full set) and maintains nine ES prone exons (http://ascot.cs.jhu.edu/ (accessed on 10 June 2022)). We observed both coding (ES ex8) and noncoding (ES ex4) ES DAS variants, but the former was rather rare (PSI < 0.1), so we disregarded it.

**Figure 12:**
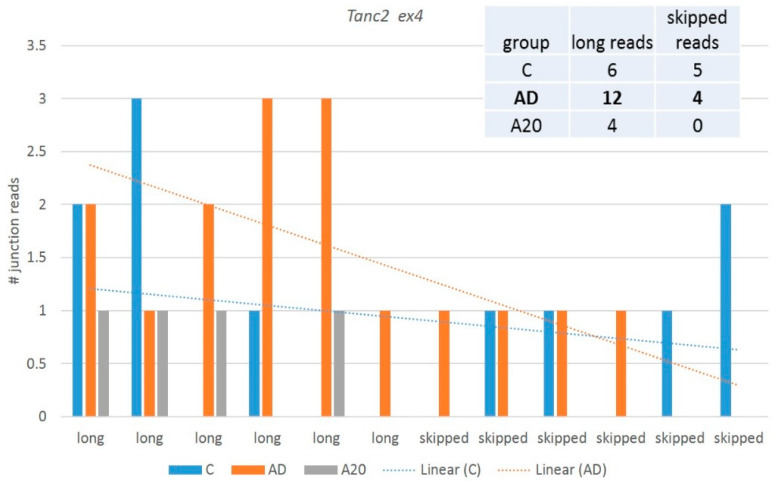
The AD and control groups differ significantly (FDR < 0.041) in the ratio of *Tanc2* splicing patterns; in particular, the AD sample maintains the maximum number of long isoforms (12 vs. 6 reads) and similar skipped ones (four vs. five; see the embedded table). The A20 group maintains the minimum number of long reads, and none are skipped, implying the overall low expression of *Tanc2* in A20.

*Tanc2* codes for a synaptic scaffold protein that interacts and co-localizes with multiple postsynaptic density proteins in dendrites to regulate the dendritic spines and excitatory synapse formation [48]. Figure 12 shows the plots of the distribution of junction reads across the samples inferred by rMATs software.

**Figure 13:**
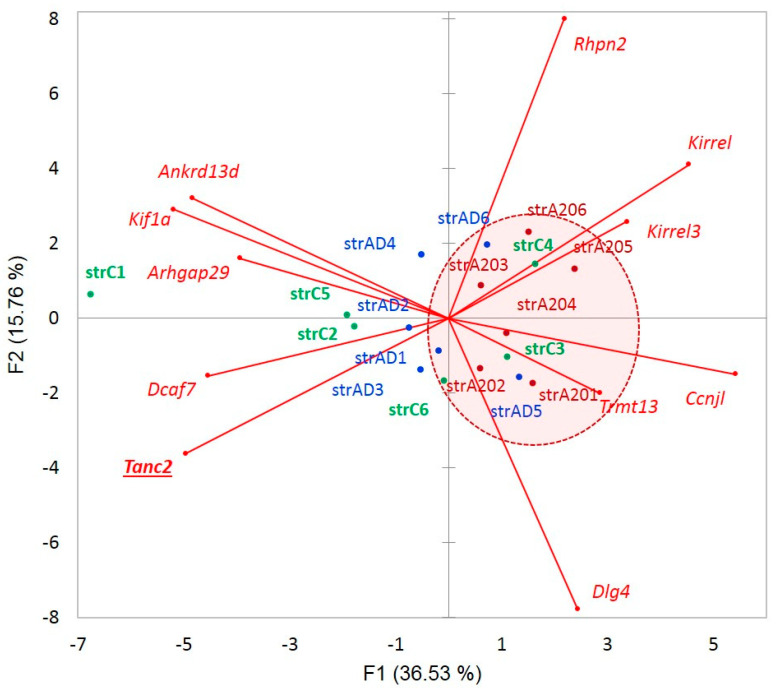
In total, 11 genes were recovered as a closest connected network environment of *Tanc2* from string-db.org (GO 0030425: ‘dendrite’, *Tanc2*, *Kif1a*, *Dlg4*, *Kirrel*, and *Kirrel3*; FDR < 0.003) shows a distinct gradient in the expression specifics of the samples. We speculate that a definite lack of a short isoform in A20 mice (Figure 2) may correlate with the attenuation of *Tanc2* expression displayed in aggressive mice: a nonrandom distribution of A20 mice in the right part of the plot (red encircled) rejects the null hypotheses with *p* < 0.0015 (binomial test).

Figure 13 shows the context of expression gradients in the network expansion of *Tanc2* gene with regard to the samples. We may see nonrandom clustering of the aggressive mice abandoning *Tanc2* expression, implying the specific dendrite contraction in the aggressive mice, which complies with the structural rearrangement of synapses in aggressive mice, earlier reported in our analysis [4]. On the contrary, we see a certain elevation in *Tanc2* expression in the AD mice, implying intensive regeneration of the synaptic architecture dynamics. Note that the apparently dopaminoceptive synaptic genes in A20 manifest a robust performance featured by *Dlg4*, *Kirrel*, and *Kirrel3* genes [4].

Figure 12 and Figure 13 suggest that although the skipped isoform expression is apparently rather background and homeostasis-mediated across the samples (Figure 12), the expression augments in AD/attenuates in A20 and the minor skipped isoform abrogates in both, leading to a significant alteration in the splicing pattern. It points to the altered overall gene network expression, seen in Figure 13. Thus, certain NMD-mediated DAS events may hint that the corresponding particular gene networks are affected.

Thus, we can infer that the synapse structural plasticity and chromatin remodeling system (Figure 11) are the most AS-enhanced in the brain-related maintenance mechanisms among the DAS ES genes.

#### 3.3.2. Nonproductive Splicing of Mitochondrial Kinase Coq8b in Aggressive Mice

**Figure 14:**
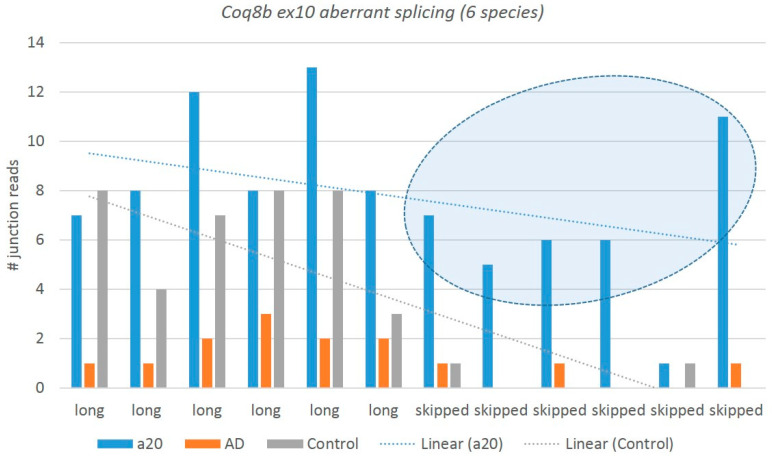
Six mice per group are shown; the ordinate shows the numbers of long/skipped junctions (Figure 2). The elevated junction numbers of *Coq8b* ex10 aberrant isoforms (exon skipping is subject to NMD) are encircled in aggressive species (blue bars). The significance of the FDR value of differentially spliced transcript ratios is 0.0028 for C_A20 comparison and 0.012 for AD_A20 (Table S2a).

We observed a dramatic elevation in the aberrant coding exon 10 skipping (94 nt) in an evolutionary conserved *Coq8b* (*Adck4*) gene (atypical kinase COQ8B, mitochondria; 25 kbp locus length), not previously annotated in any public databases, expressed specifically in the aggressive mice (Figure 14). It follows the overall increase in the *Adck4* expression in the aggressive group (9 FPKM) as compared with the AD/control (8.7 FPKM), not reaching a statistical DEG level. The exon 10 skipping leads to a premature termination codon (PTC) following an NMD. The expression of aberrant isoforms is vanishingly small in the control mice as compared with the aggressive ones: two junctions vs. 36 in the A20 mice (Figure 14). It may be a consequence of the *Coq8b* overexpression in A20. Note that this exon comprises at least three conserved missense variants: rs3388875016, rs3388890121, and rs13460395 (European Variant Archive (EVA) release 3).

Along with the *Coq10a* and *Coq10b* genes, the *Coq8b* gene facilitates the biosynthesis of coenzyme Q10 (ubiquinone-10), an essential lipid-soluble electron transporter for aerobic cellular respiration [49]. In mitochondria, coenzyme Q10 plays a key role in oxidative phosphorylation. Coenzyme Q10 is also involved in pyrimidine synthesis and ATP and GTP production in the cell [49]. In the cell membranes, coenzyme Q10 acts as an antioxidant, protecting cells from the damage caused by unstable oxygen-containing molecules (free radicals), which are known to cause disorders due to primary coenzyme Q10 deficiency [49].

In mammals, the respiratory complex I (NADH: ubiquinone oxidoreductase) of the mitochondrial respiratory chain has 38 subunits; the NMD mechanism maintains the homeostatic balance along with a wide range of coding isoforms [50,51]. It catalyzes the transfer of electrons from NADH to coenzyme Q10 (*Coq10*), which translocates protons across the inner mitochondrial membrane.

Herein, we report a new *Coq8b* noncoding isoform with skipped exon 10 as a highly consistent NMD event in the DS of the aggressive group (Figure 14). Two redox multisubunit complexes, namely, complex I (*CoI*) and coenzyme Q substrate synthesis (*Coq10*), feature highly dynamic RNA SFs at the stage of RNA processing [51].

**Figure 15:**
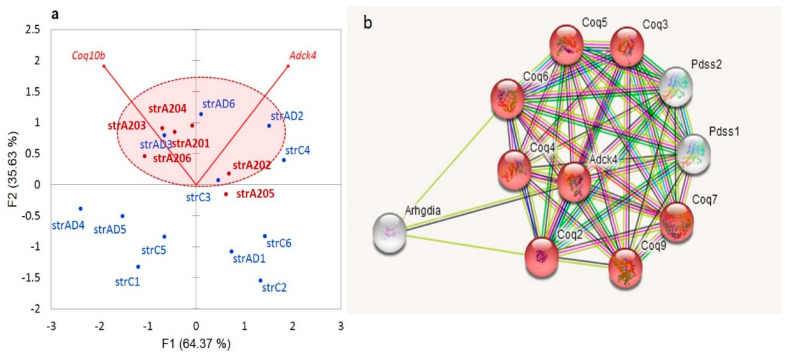
(**a**). The expression rate of *CoQ10b*, along with *Adck4* (*Coq8b*), is elevated in the A20 group (encircled; random probability of five of six A20 mice located in the upper half is *p* < 0.01 based on a binomial test). (**b**). The gene network for *Adck4* (*Coq8b*) according to string-db.org resource. Red shaded nodes relate to GO: 0005743: Mitochondrial inner membrane (8 of 435 genes; FDR < 2.2 × 10^−9^).

We observe an elevation in the redox-related genes in the A20 mice, as is shown in Figure 15a. Apart from the *Coq10* genes, the synthesis of ubiquinone requires a set of other mitochondrial genes (Figure 15b).

### 3.4. Assessment of the Overall AS-Mediated Proteome Plasticity in the DS

Notwithstanding a small number of DAS genes (in total, 91 DAS; Table S4d), we addressed the overall pool of AS ES coding/NMD events (about 7000 per comparison) by filtering (see Methods) to assess the protein-coding competent events. We performed the procedure for both the exon triplets (long isoform) and the exon duplets (skipped isoform). When assessing the coding events, we found out that the number of ES events with either/both coding isoforms account for approximately a half (Table 2 and Table 3) of the total ES events listed in Table 1 due to the UTR ES exons. We also observed a range of three-set exons employed in multiple tandem splice sites (TASS; ‘bleeding’ 3′/5′ exon flanks; [52]; the mode is three overlapping reads per sample), which, as a rule, display a low isoform ratio and disrupt ORF.

We filtered those out by a threshold on the ES junction coverage to be more than five reads per ES event. Finally, we computed the corrected number of the whole transcriptome coding AS ES events, listed in Table 4.

**Figure 16:**
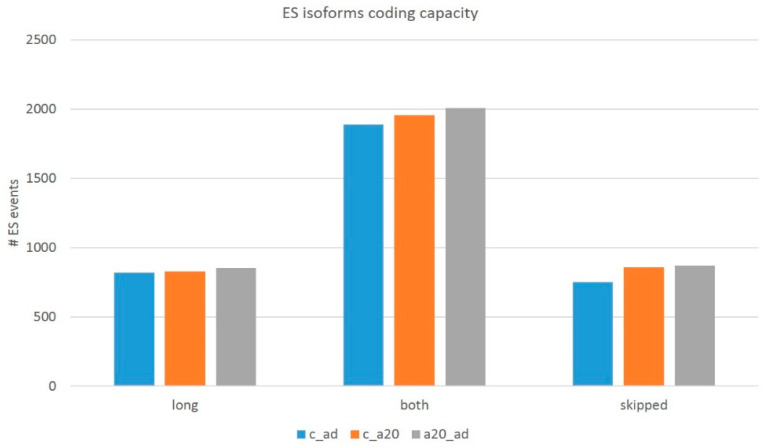
Table 4—inferred plot on the protein competent ES events implies consistency in the overall distribution: only half of the ES events maintain both long and short coding isoforms. Abbreviations: long, long coding isoform alone; both, both isoforms are coding; and skipped, skipped coding isoform alone.

In this way, we ascertained the transcriptome-wise distribution of two basic types of ES events (Table 4 and Figure 16): (a) AS2 genuine alternative coding isoforms (category of ‘both’ isoforms) and (b) NMD-associated regulatory ES events, when either isoform (long/skipped) is noncoding. Further, we will pursue to ascertain the functional categories of the genes with AS2-coding ES events and NMD-associated ones.

#### 3.4.1. Assessing the Distribution of the Major Splicing Isoforms in the Gene Pool

**Figure 17:**
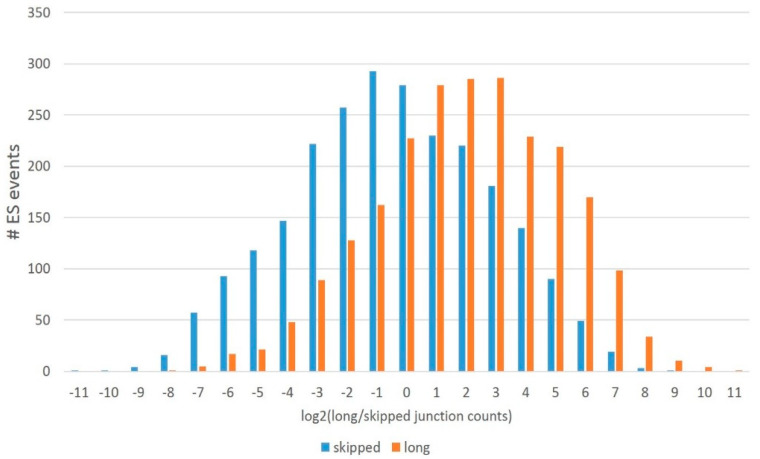
Distribution of the expression log ratio of long/skipped coding isoforms based on junction counts (abscissa): 0 corresponds to long and skipped isoforms equally expressed; positive numbers signify preferential long isoform expression; and negative numbers point to skipped isoform preference.

Based on the protein-competent isoforms, we assessed their expression throughout the ES skipping data across all sets. We found out that a long isoform is generally preferable in the DS proteome (Figure 17) since a skipped isoform average/mode is approximately log2 = –1, while a long isoform maintains the mode around log2 = 2 (Figure 2), implying a threefold higher long isoform expression average.

#### 3.4.2. Assessment of Gene Families for Coding AS Events

We also assessed the statistics of most ES events across the abundant gene families listed in Table 5.

While the SLC family is the top by the number of genes, we may see that the highest ES skipping rate families (number of ES events per gene) are *Ank*, *Cam*, *Kid*, and *Myo*.

Another statistic of interest is the replicability of the ES events across the C_A20 and C_AD compared groups (Table 6). We observed rather consistent numbers of the coding ES events per family therein.

While the AS plasticity analysis of the overall gene families in the transcriptome is beyond the scope of our paper on the analysis of DAS genes, it emphasizes the excess of ES events prevalent in the neuron-specific genes.

#### 3.4.3. Gene-Wise Comparison of the Overall AS Rate

Based on three pairwise comparisons, we outlined the genes that manifested the maximum protein coding variation starting from at least four AS ES events (AS2, both isoforms coding; Table S6a) and those that manifested the most NMD-related ES events (either isoform is coding; Table S7a) yielding at least two NMD isoforms per gene. We termed the lists of these genes as ‘AS2/NMD ES enriched genes’.

There were no NMD abundant genes with more than five NMD ES events per gene (Table S7a), while AS2 abundant genes displayed the top 12 coding isoforms per gene (Table S6a). Therefore, we used a lower threshold of four isoforms per gene for the AS2 samples and two isoforms for the NMD samples.

Note that all three lists of comparisons (C_A20, C_AD, and AD_A20) are 82% identical, reflecting that the selected top-ranking genes maintain AS as a key plasticity factor in their working routine (Table S5a).

#### 3.4.4. AS2 Genes Top Enriched with ES Events (Both Isoforms)

**Figure 18:**
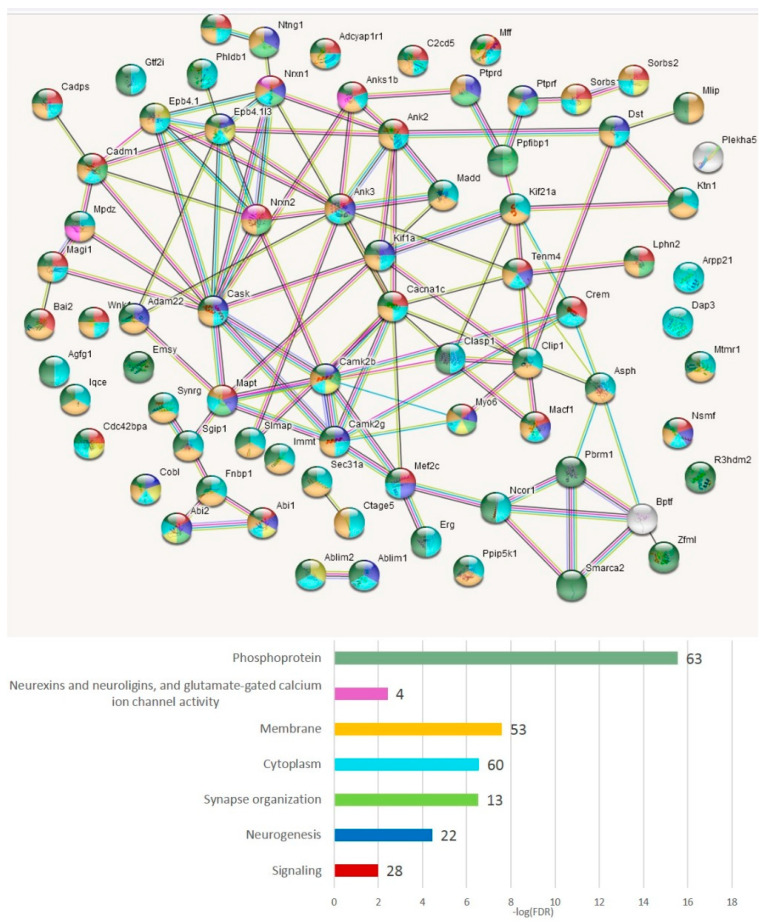
String-db.org connectome emphasizes that the AS2 (coding) ES abundant genes (75 entries; Table S6e) are highly interconnected (PPI enrichment, FDR < 1 × 10^−16^), and non-randomly express specifically in membrane/cytoplasmic compartments. Full GO annotation is available in Table S6f. An expanded network version of the figure can be found in Table S6f.

Table S6a–d show the lists of genes and annotation of the GO terms per comparison for readers to scan, but we chose the merged gene list as the main target. In total, 75 genes (Table S6e and Figure 18) manifest the maximum protein coding AS2 ES events per gene (from ten to four, Table S6a). We applied the GO enrichment annotation routine (string-db.org) to elucidate the enrichment of the major functions in this pool (Table S6f). We obtained a highly interconnected network (Figure 18) by string-db.org suite with the number of edges of 20 vs. the expected 7 ones (*p* < 1 × 10^−16^). Note that 63 genes were annotated as ‘phosphoprotein’ (FDR < 1.7 × 10^−14^; Figure 18). The ‘synapse’ termed genes comprised 35 genes (FDR < 5.3 × 10^−18^; Table S6f).

As is evident from Figure 18, the most connected are the synaptic and other membrane-associated genes, including NOVA SFs regulated ones (Table S6f). Inferring from the GO annotation, the vast majority of AS2 ES-rich genes are related to the membrane/cytoskeleton (Table S6f).

#### 3.4.5. Genes with the NMD-Related Top Abundant ES Events

Several ES NMD exons present in a single gene may imply the instantiation of NMD events by certain SFs, pointing at multilayer gene regulation in various environments. Alternatively, several synchronically manifested NMD-associated joint ES events may lead to an overlapping protein product but, as our preliminary analysis showed, we observed no ORF coding compensation shift within a vicinity of an NMD event; as a rule, it disrupts the ORF by stop codons within a single exon following an ES NMD event. Moreover, multiple NMD exon-containing genes are not abundant; we found only approximately 50 genes with the number of ES NMD > 2 (however, 221 genes with the number of ES NMD = 2) in our three comparisons (Table S7a). Still, the point of several gene maintaining conservative/consistent NMD exons requires further investigation.

**Figure 19:**
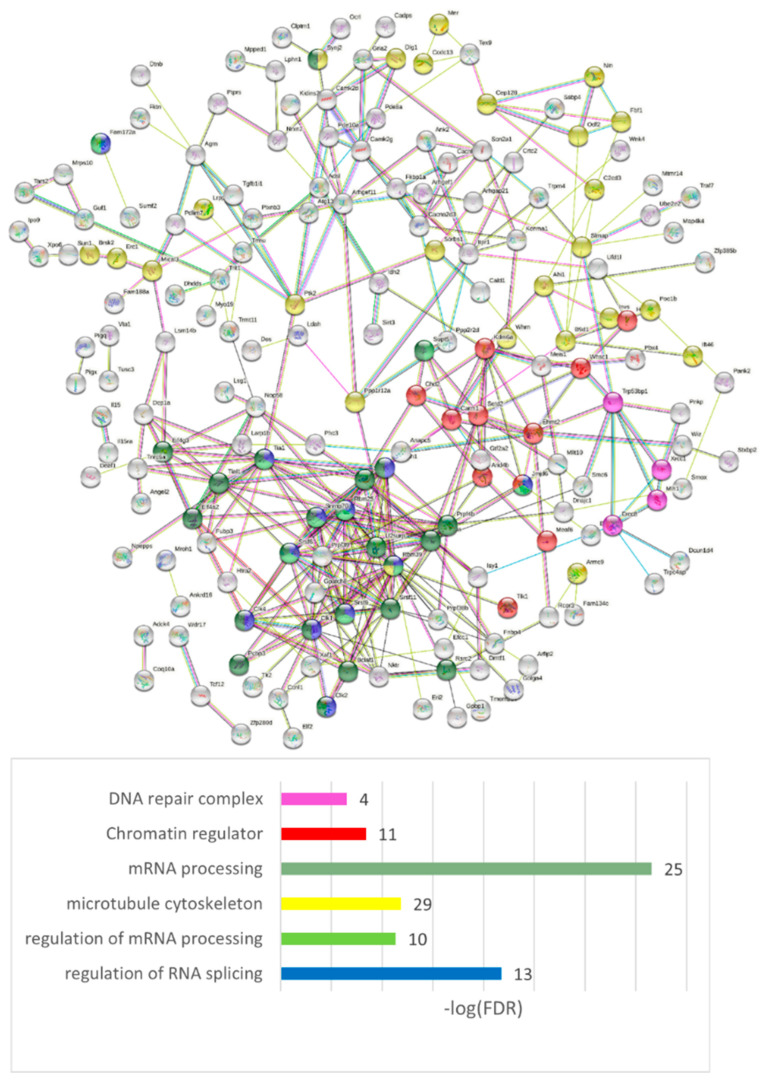
NMD-type ES event abundant genes (Table S7b) were mostly assigned to the nuclear compartment. Full GO annotation is shown in Table S7c. See Table S7b for an expanded network version of the figure.

We performed the GO annotation of 271 NMD ES abundant genes merged across three comparisons (Table S7a), listed in Table S7b (Figure 19 shows the connected network), and found a striking discrepancy with the AS2 type ES abundant genes: most of them were nuclear genes.

Our analysis suggests that the NMD-related events are predominantly associated with DNA/RNA processing events. We observed the NMD ES abundant events in the nuclear genes to be highly nonrandom (GO term: ‘Nucleus’, 92 genes; FDR < 9.2 × 10^−9^ vs. ‘Cytoplasm’, 117 genes; FDR < 3.4 × 10^−4^; Table S7c, ‘Compartment’), while the AS2-related splicing mode is the most represented in cytoplasmic/membrane/synapse genes (GO term: ‘Nucleus’ insignificant enrichment (FDR > 0.05); ‘Cytoplasm’, 46 genes; FDR < 1.8 × 10^−6^; Table S6f, ‘Compartment’). We assert that a significant part of the genes uses both the AS2/NMD means of regulation in both compartments, but the distinct compartment priority is statistically significant.

**Figure 20:**
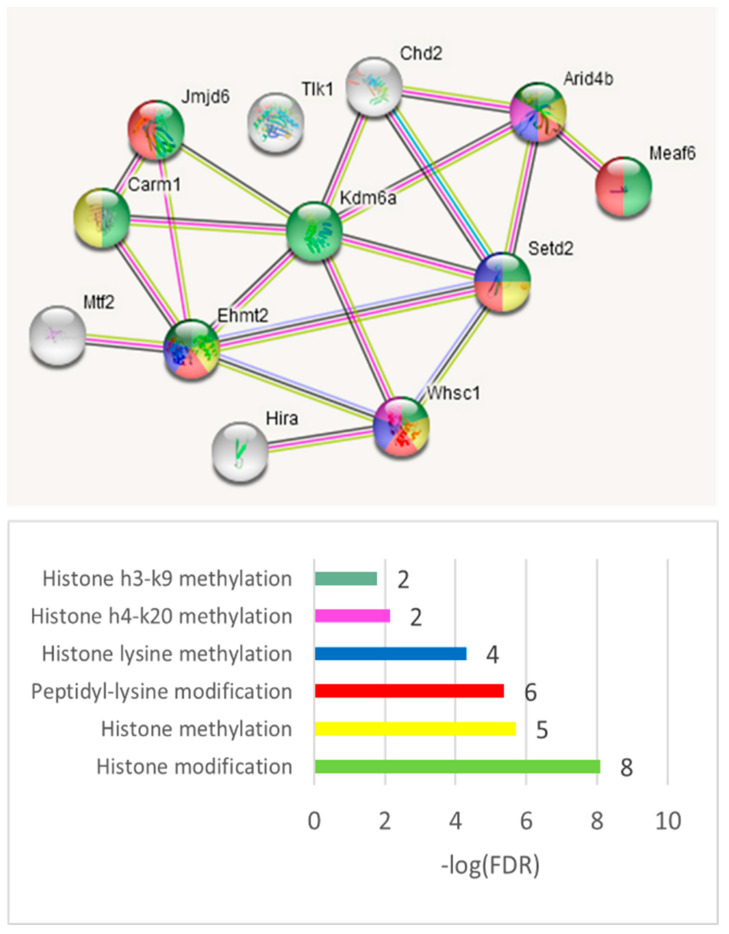
The 11 ES abundant closely connected genes with NMD exons from Table S7c (string-db.org; KW-0156: ‘Chromatin regulator’; 12 genes).

We observed many coding events in the chromatin regulators (see DAS genes in Figure 11). As for the NMD ES events, we found that the histone methyl/acetyltransferases particularly intensely utilize NMD, implying a tight homeostatic coordination between the histone modification complex subunits. Figure 20 shows 11 genes from the ‘chromatin regulator’ GO category (Figure 19, ‘Chromatin regulator’; Table S7c) gene network enriched in ES NMD events.

#### 3.4.6. ES Abundant Genes Splicing Factor (SF) Network

**Figure 21:**
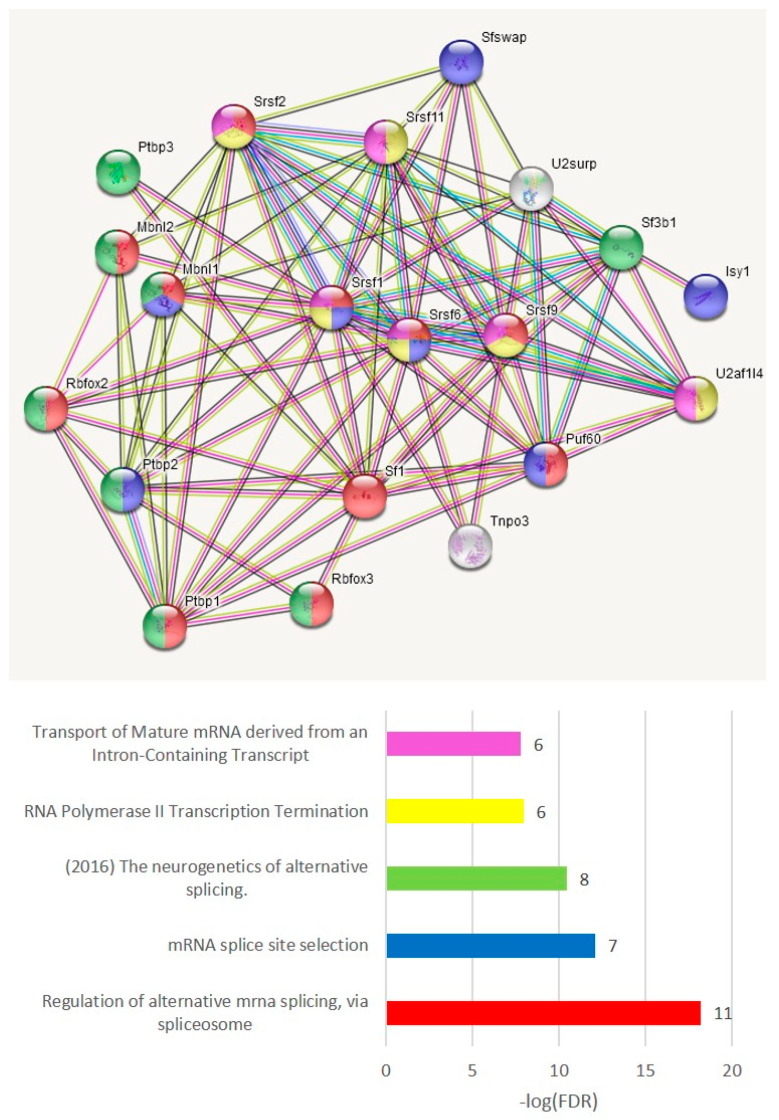
Network of 21 SF genes abundant in the AS2/NMD ES events observed in our samples (Table S8a) outlined by string-db.org. Color code corresponds to the bar plot.

To grasp a view of the SF specific preference across the groups, we elucidated the SF network of the ES abundant genes (Tables S6a and S7a), shown in Figure 21. This suggests a tight auto- and cross-regulation of SFs and RBMs, as is earlier described [53,54,55,56,57]. From notable examples, polypyrimidine tract binding proteins (PTBP1–3) are reported for intricate self- and cross-regulation during the neurogenesis and neuronal differentiation [56,58] along with microglial/endothelial immunogenic competence [56,57,59].

**Figure 22:**
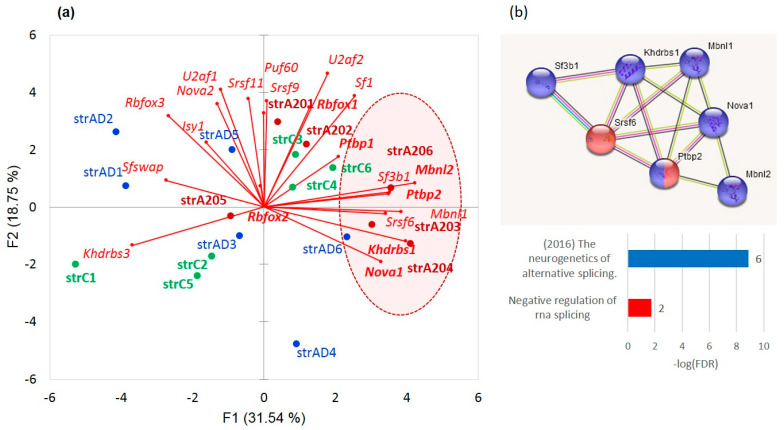
(**a**). Distribution of the SFs across the groups of mice. We observed five out of six A20 samples in the right side of the plot (random chance *p* < 0.016 based on a binomial test), implying consistent elevated expression of particular SF subsets for this group. Boldfaced are the SFs mentioned in [56], which maintain *Grin1* and *Dlg4* (*Psd95*) isoforms were tuned up (encircled with red shaded oval). (**b**). The coordinated cluster of the SF factors encircled in (**a**) represents the annotated coordinated SF network reported, in particular, in [56].

As is evident from Figure 22, 21 splicing factors (Table S8b) are distributed in a group-specific manner: AD subjects (blue) tended to locate in the left part of the plot, while aggressive ones clustered in the right side (red). The control mice (green) split into two subgroups (states), one with elevated Khdrbs3 (SLM2) and the other with elevated expression of *U2af2*, *Sf1*, *Rbfox1*, and *Ptbp1* SF clusters. *Srsf9*, *Srsf11*, and *Puf*60 (poly-U binding factor) expression is nonspecifically relative to A20 (right plot part), while AD (left plot part) gradient lines are located close to the ordinate. The expression of *Rbfox2* is nonspecific with regard to the distribution of the groups.

We delineated the cluster of seven encircled genes (*Sf3b1m*, *Khdrbs1* (*Sam68*), *Srsf6*, *Nova1*, *Mbnl1*, *Mbnl2*, and *Nova1*), in Figure 22a, featuring the aggressive mice, and separately shown in Figure 22b, using the string-db.org suite. Based on the report [56] describing these genes, we speculate that they maintain intense AS tune-up of postsynaptic *Grin1* and *Psd95* (*Dlg4*) genes of the medium spiny neurons (MSNs). We have earlier ascertained that these gene isoforms tune up, playing a pivotal role in cAMP-mediated D1/D2 dopamine signaling pathways, which are the most intensive in the MSNs of aggressive mice [10]. Both genes maintain an elevated expression, specifically in aggressive mice although in a statistically insignificant manner (*p* < 0.1, binomial model). Moreover, the *Grin1* and *Psd95* expression is significantly correlated in our samples (*r* = 0.741; *p* < 0.0004; df = 17).

Note that while [56] addressed the cortical neurons in their study, they reported that the skipping of *Psd95* exon 18 was subject to NMD. We also observed nonproductive skipping of the same exon but with a low frequency (PSI < 0.1). In addition, they also emphasize that the SFs analyzed herein actively interact with the other RNA binding proteins, as was shown in our earlier publication on the same data ([4], Figure 12 therein).

As was earlier reported [60,61], all the ES events in *Grin1* are coding, with three basic isoforms (sorted in a descending order by the transcription rate in DS [10]): NM_001177656, NM_008169, and NM_001177657. We observed that these isoforms were expressed phase-specifically in the cAMP-mediated cascade of D1 MSNs, as noted in an earlier publication [10].

## 4. Discussion

We performed RNA-seq analysis of aggressive (A20), aggression-deprived (AD), and control groups evaluating their relationships. After the previous elucidation of the networks of DEGs [4], we concluded that a high dopamine uptake accompanied by endogenous opioid genesis in A20 resulted in a profound alteration in the DS metabolome leading to over 1000 DEGs in A20 as compared with two other groups. Accordingly, the comparison of the AD vs. control group yielded only 62 DEGs [4], implying considerable recovery of the DS transcriptome after 2 weeks of fighting deprivation in the AD group. Thus, most of the DEG changes are related to many gene circuits outlined for the AD_A20 GO annotation.

This was not the same for the DAS analysis because of an intensive AS ES revealed in both affected groups. The most significant DAS genes were observed in the AD group (vs. control): *Ppp1r12b*, *Nfix*, *Thoc5*, *Hdac7*, and *Rnf144a* maintain the top significant DAS FDR < 1 × 10^−5^ (Table S2a). The A20 vs. AD group displayed the smallest number of highly significant DAS events (Table S2a), implying that the stress-mediated DAS genes were common in both (e.g., *Hdac7*). Still, the most extended DAS ES list is characteristic of C_A20, implying distinct features of the A20 performance state reported in [4]. In particular, the expression profiles of the corresponding DAS-related networks (the PCA plots throughout the text) show that DAS genes simultaneously decreased their expression in the A20 group cluster (Figure 8, Figure 9 and Figure 11, etc.). It implies the AS events association/mediation with expression downturn of many gene networks in the A20 group, as previously reported [4].

An intensive chromatin rearrangement in the amygdala, in particular, histone (de)acetylation, was reported in connection with alcoholism and anxiety behavior upon withdrawal [62]. Specific significant DAS ES events are observed in chromatin factor *Hdac7* (FDR < 2.3 × 10^−5^ in C_AD, Table S2a) in both A20 and AD relative to the control. Furthermore, we observed four other linked chromatin remodeling factors (*Ezh2*, *Rarb*, *Chd2*, *Brdt*, and *Scrap*; Table S3b) manifested selectively by both affected groups (Tables S2a and S3c). While we would expect chromatin rearrangement machinery being employed as a mean of the silencing/activation of stress responsive gene networks, we herein underscore that chromatin machinery widely employs the AS ES switching as a mean for performing the corresponding histone/chromatin modifications upon a consistent stress response specifically in the brain/DS, which has not been explicitly reported before, as far as we know. Another point is that HDACs, in particular, may interact with spliceosomal and ribonucleoprotein complexes and affect the acetylation states of the splicing-associated histone marks and splicing factors, thereby modulating the splicing outcome, as recently proposed [63].

**Figure 23:**
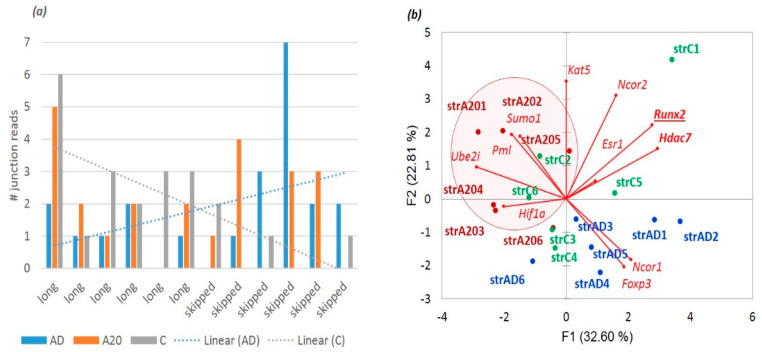
(**a**). Alteration of the *Hdac7* major isoform in the AD mice from a long to a short one. (**b**). The plot of PCA expression profiles of the *Hdac7* closest connectome (10 genes; source: string-db.org) displays the clustering of both the A20 and AD mice in distinct antagonistic compartments, both avoiding *Hdac7* expression. AD preferable genes are *Ncor1* and *Foxp3,* and A20 are *Sumo1*, *Pml*, *Ube2i*, and *Hif1a. Runx2* (runt-related transcription factor 2) is a DEG in the C_A20 comparison [4], which is highly correlated with *Hdac7* (*r* = 0.897; *p* < 0.00014, df = 17).

Figure 23a illustrates the fact that *Hdac7* maintains a significantly elevated minor isoform both in the A20 and AD samples, implying attenuation of the *Hdac7* catalytic activity, which is also supported by Figure 23b, and implies the attainment of active an chromatin state for the neuron structure recovery gene networks in AD mice [4].

Other highly significant and repeatedly confirmed (Table S4b) DAS ES genes are ankyrin *Anks1b* (FDR < 2.3 × 10^−4^) and *Ppfibp1* (*Znt9*, FDR < 2.8 × 10^−4^; Table S2a). A string-db.org analysis confirmed three particular gene networks, shown in Figure 7. Note that active chromatin regulation (observed in A20-mice; Figs.19, 20) NMD instantiation in the neuronal cell types was first reported in [47] and has been proven to be conserved across the mammalian clade therein. We report that besides the NMD-associated ES events in chromatin regulation, we observed the chromatin rearrangement complex (SWI/SNF) contains coding ES events for the histone modification enzymes, implying the active chromatin rearrangement in aggressive mice (Figure 18 and Table 5b).

Thus, the DAS analysis of the coding ES events suggests that the glutamatergic synapse and chromatin rearrangement gene networks actively exploit the AS machinery, providing the means of plasticity to these complexes (Figure 4). We also observed the employment of PKA-associated *Grin1* isoforms, reported earlier in our paper [10].

**Figure 24:**
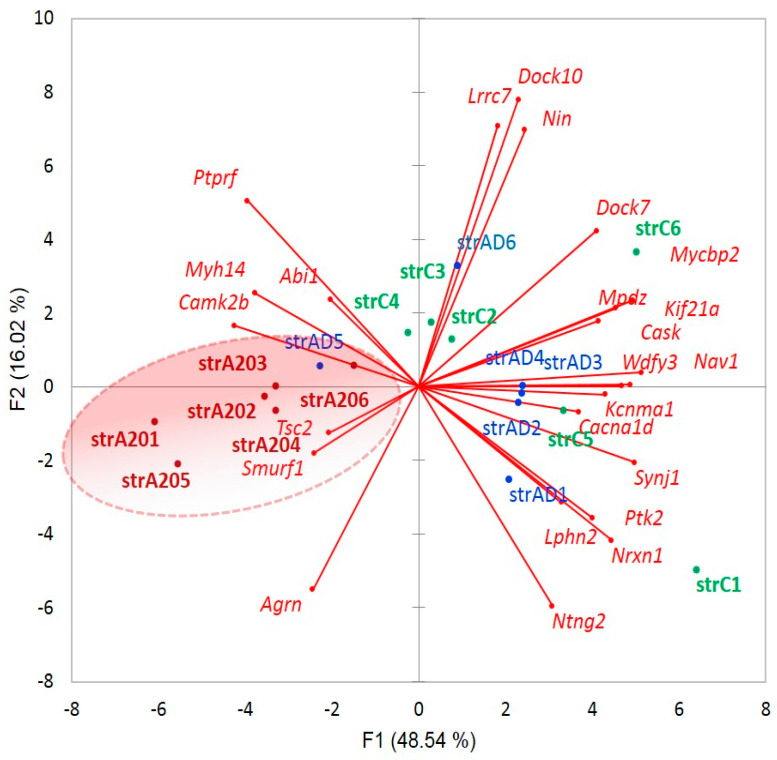
PCA plot of 24 genes belonging to the ‘neuron projection’ GO category with abundant AS ES events built using their expression profiles (Tables S6 and S7). The synaptic architecture of ES abundant genes is distinct between A20 and the two other groups. The left gene cluster is the GO annotated for ‘glutamatergic synapse’ (three genes, FDR < 0.01), ‘axon’ (six genes, FDR < 1 × 10^−5^), and ‘growth cone’ (five genes, FDR < 6 × 10^−6^), and the right cluster, for ‘intrinsic component of presynaptic active zone membrane’ (four genes, FDR < 4.2 × 10^−6^). A nonrandom location of the A20 mice number (6) in the left part of the plot maintains *p* < 0.0014 (binomial test).

We selected 24 genes with abundant protein-coding AS ES events for assessing the ‘neuron projection’ GO category (Tables S6 and S7). We found that the overall architecture of the AS synapse genes with intensive expression in A20 vs. AD and C significantly differs between the groups (Figure 24).

By assessing the overall proteome variation, we infer that at least half of the AS ES coding events are capable of maintaining both isoforms as coding. In addition, this revealed that half of the coding events (~1800 genes, Table 5) manifest an NMD-related AS event, leading to ORF disruption and subsequent mRNA degradation.

We found out that the AS2 (both coding isoforms) ES abundant genes are mostly related to the membrane/cytoplasm-associated genes (Figure 24 and Table S6a–c), which was also earlier reported [64]; therefore, the authors used the ribosome-engaged transcripts to ensure the RNA coding competence.

The SWI/SNF superfamily-type complex of chromatin modifying/remodeling enzymes *Bptf*, *Smarca2*, and *Pbrm1* (Table S6a–c) also use a dynamically altering ratio of coding isoforms, namely, at least four distinct AS2 ES events are observable in *Smarca2* and six AS2 events in *Pbrm1*, closely associate with it (Table S6a and Figure 18). *Pbrm1* (syn: *Baf180*, *Hpb1*, *Smarch1*, and *Brg1)* was shown to modify AS by interactions altering the RNA binding factors with nascent RNA [65], as well as *Smarca2* itself [66], making the SWI/SNF complex an auxiliary splicing machine actively involved in the splicing outcome while being regulated by AS itself, specifically in the neurons [67]. We have not seen many publications on the SWI/SNF AS competence yet; however, we have regularly observed that it maintains coding AS ES events in our samples, in particular, in hippocampus samples (personal observation). In this study, we observed multiple coding isoforms of both genes (Table S6).

The NMD-associated transcripts have been long reported in auto- and cross-regulation of the SFs in a range of the papers describing complex cascade relationships between these factors [53,54,55,58,68,69]. Our method of elucidating the NMD-related ES events have allowed us to assess the properties of the genes maintaining the multiple (more than one) variants of these events (Table S8). Strikingly, we have observed that the major NMD events are related to nuclear genes (full GO annotation charts for the NMD ES abundant genes are shown in Table S7), which was also reported in [47]. The RNA processing proteins, including spliceosomal components, were nonrandomly enriched. In addition, ubiquitin-like (*Ubl*) conjugation (*E2S*) genes were also enriched in NMD ES events.

**Figure 25:**
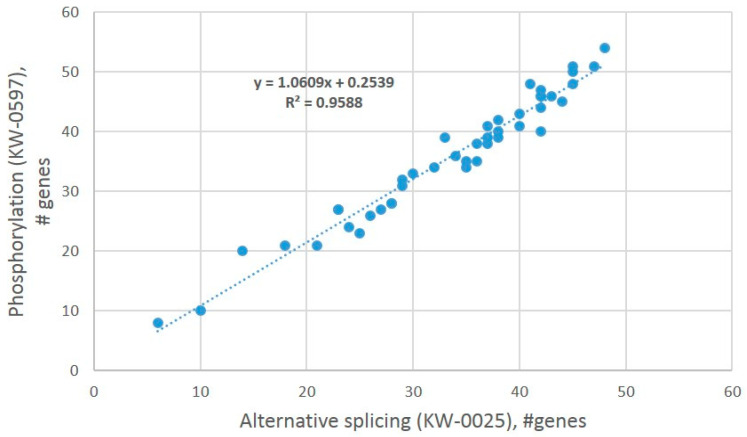
Significant co-occurrence (*p* < 1.0 × 10^−30^, df = 43) of AS vs. phosphoprotein GO keyword categories (UniProt) unveiled by a regression plot. GO source: string-db.org; random gene sets subsampling from merged ES abundant lists (Table S7a–c).

The analysis of the GO annotation of ES AS abundant genes (Tables S6 and S7) allows us to state that the majority of alternatively spliced proteins are phosphoproteins (see, e.g., Figure 18), underscoring AS coupling with reversible phosphorylation. The latter is the major signal transduction means in the DS, including the core signaling of cAMP-mediated dopaminoceptive response [4]. By random sampling of AS-rich gene subgroups and annotating them for ‘AS’/’phosphoprotein’ GO terms, we confirm that ES AS abundant genes, as a rule, also maintain reversible phosphorylation, as suggested by regression analysis (Figure 25).

Energy homeostasis also widely employs ES NMD AS in mitochondria [51]. The ADCK4 (COQ8) kinase protein resides within the inner membrane of the mitochondria and is associated with ATP binding. Notably, we have observed abundant ES NMD-related instances in mitochondrial *Coq8* exon 10 skipping not reported before, featured in aggressive subjects along with the overall elevation in *Coq8* expression (Figure 9). This underscores the NMD machinery being effective even in a very optimal evolutionary conserved energy metabolism (oxidative phosphorylation) pathway, yet may be aberrant according to a recent report [70]. The energy issues due to mitochondrial dysfunction in stress related abnormalities consistently reported previously imply the unproductive splicing in *Coq8* observed in our study may affect energy homeostasis.

These facts suggest a certain preference in the NMD-associated vs. AS2 events in a compartment-specific manner. As for the functional consequences of our work on the DS of mice in the social stress model, we emphasize the importance of the interplay between dopamine and glutamate receptors in MSNs [4,71]. Glutamate receptors are the most AS-effective neural genes in terms of (a) the lack of NMD-related events and (b) the presence of two major isoforms in the NMDA subunit gene *Grin1*, mainly differing in the glycine affinity in the N-terminal domain (ex5 skipping). Notably, *Grin1* functional splicing activity has been widely discussed since 1992 [72,73,74,75].

## 5. Conclusions

Several publications emphasized the exon-centered AS program as a way of gene complexity evolutionary expansion in mammals by both the exon shuffling and exon splicing variations [30,76]. This highlights the most intensive splicing rate in the brain transcriptome [69] upon the emergence of a brain-specific arsenal of SFs regulating the in/out splicing of specific exons [5]. Thus, a special exon splicing machinery has emerged in the course of rapid animal evolution in the form of brain-specific SFs maintaining the neurospecific gene regulation in temporal and spatial manners, often employing cross- and self-regulation by means of NMD as well as providing an additional coding neuroplasticity layer [5].

We assessed the global splicing AS ES specifics in the dorsal striatum transcriptome. Our genome-wide transcriptome AS ES analysis outlined that a half of the ES events are located in the 5′/3′UTR (Table 4), while the associated coding ES variants maintain half of the alternative isoforms containing premature stop codons subject to NMD. We also observe that the majority of ES events are related to housekeeping regulatory machineries affecting the transcription rate, including the spliceosome, RNA processing, chromatin rearrangement, mRNA export, transcription factors, and many others. In addition to nuclear-associated genes, the membrane/synapse/cytoskeleton-associated genes manifest another group that are highly enriched with ES events genes. Splicing is intensively employed in the brain-specific gene families, such as *Slc* and *Fam* (Table 6).

We found that NMD-mediated splicing is prevalently characteristic of nuclear genes, including the spliceosome. Another machinery that intensively utilizes splicing specifically in the brain is the chromatin remodeling system, as reported earlier [16]. The key components of the chromatin rearrangement complex, including SMARCA and ACD7, intensively use alternative coding isoforms, as is confirmed by our study. We have also shown that the TF programs are actively switched in the course of aggressive adaptation [4] by means of intensive chromatin remodeling. We would also like to assert that numerous AS2 coding alterations are often related to the expression rate and the metabolic activity/binding strength, as it follows from the DAS vs. DEGs comparison, thus also addressing quick homeostasis adjustments/transitions.

The majority of coding AS2 ES dynamics takes place in the membrane proteins, including synaptic genes. The AS2 genes subject to ES events are also significantly overrepresented by phosphoproteins. We thus witness the AS ES-rich genes undergoing posttranslational modification by the (de)phosphorylation machine, implying a high flexibility of these genes with respect to both phosphorylation and splicing encoding systems employed in their temporal/spatial tune-up. It is especially evident for the genes involved in the cAMP-mediated signaling cascade in the DS MSNs, which actively utilize both phosphorylation machinery (DARPP-32 and others, switching phosphorylation marks), as well as D1 MSNs phase-dependent AS switching of major *Grin1* isoforms to enable agent-specific (D1/PKA) switching [4,10]. We hypothesize that the linkage of AS to phosphoproteins may also imply that some of the phosphorylation sites can be excised by AS ES, which is the case of the DARPP-32 protein truncated isoform t-DARPP with exclusion of the key PKA phosphorylation target position 34 [4].

As a key finding, we report significant elevation of SF expression activity in the A20 group, which is apparently linked to the enhanced performance on the cAMP cascade.

## Figures and Tables

**Table 1:** Distribution of the statistically significant (FDR < 0.11) differential exon splicing events across three comparisons by rMATs.

	DAS Genes Significantly Differing (5′/3′UTR ES Included) between Corresponding Samples (DAS); Table S2a	Unique Coding DAS ES Events; Table S3a	Alternative ES Events in Total
Control vs. A20	82	46	7264
Control vs. AD	65	36	6781
A20 vs. AD	68	36	7378

**Table 2:** Distribution of AS2/NMD coding ES events.

	AS2 *	NMD *	Total
C_AD	24	12	36
C_A20	27	19	46
AD_A20	21	15	36
Total unique genes (after merging)	59	32	91

* AS2, both isoforms are protein coding, and NMD, either one (long/skipped) is protein coding.

**Table 3:** Number of the AS2 DAS ES common events between three mouse groups (Table S4b). The overall DAS numbers in the corresponding pairwise comparison are boldfaced.

Group Pairs	C_A20	C_AD	AD_A20
C_a20	**82**	23	22
C_ad	23	**65**	20
AD_a20	22	20	**68**

**Table 4:** Distribution of total consistent coding ES events in pairwise comparisons.

	Type of Coding Isoforms			
	Long	Both	Skipped	Total
C_AD	818	1888	751	3457
C_A20	827	1955	857	3639
A20_AD	852	2006	869	3727

**Table 5:** Distribution of the top ES events across gene families sorted in descending order of the number of ES fields. Statistically significant DAS FDR for a particular gene from the family (if any) is boldfaced.

Family	Sample	#Genes	#ES	Min (FDR)	Description
Slc	Slc2a6	35	41	0.800464	Solute carrier family 2
Map	Map1s	18	30	0.72088	Microtubule-associated protein
Arh	Arhgap21	18	29	0.942245	Rho GTPase activating protein
Fam	Fam168b	16	25	**0.017541**	Family with sequence similarity
Zfp	Zfp930	15	21	0.163404	Zinc finger protein
Ccd	Ccdc187	14	14	**0.002809**	Coiled-coil domain containing
Eif	Eif2d	13	14	0.179903	Eif (transcription factor)
Tmem	Tmem131l	13	15	1	Trafficking protein particle complex subunit
Ank	Ank2	12	33	**0.047135**	Ankyrin 2, brain (Ank2)
Ptp	Ptpn9	12	24	0.121426	Protein tyrosine phosphatase
Usp	Usp37	11	13	0.200071	Ubiquitin-specific peptidase
Cam	Camk2b	10	26	1	Calcium/calmodulin-dependent protein kinase II
Kif	Kif23	10	21	1	Kinesin family member
Cac	Cacul1	9	18	1	CDK2 associated cullin domain 1
Kcn	Kcnn1	9	16	1	Potassium calcium–activated channel subfamily N
Myo	Myo9b	9	18	1	Myosin IXB, actin-associated
Rab	Rab11a	9	10	1	RAB3 GTPase activating protein catalytic subunit 1
Tra	Traf7	9	11	1	TNF receptor associated factor 7 (TRAF7); trafficking protein particle complex subunit (TRAPPC1,4,6,9-13)

**Table 6:** Gene families (defined by the three first letters of the gene name) with the top number of coding ES skipping events for C_A20 and C_AD comparisons. The statistically significant events in a specific gene of the family in a particular comparison are boldfaced.

	C_A20					C_AD		
Family	Sample	#Genes	#ES	Min (FDR)	Sample	#Genes	#ES	Min (FDR)
Slc	*Slc30a9*	*32*	*37*	*7.800 × 10^−5^*	*Slc2a6*	35	41	0.800
Fam	*Fam135a*	19	29	0.885	*Fam168b*	16	25	**0.018**
Arh	*Arhgap21*	17	31	0.563	*Arhgap21*	18	29	0.942
Ccd	*Ccdc187*	16	19	1.000	*Ccdc187*	14	14	**0.003**
Map	*Map1s*	15	27	0.311	*Map1s*	18	30	0.721
Zfp	*Zfp930*	14	20	0.193	*Zfp930*	15	21	0.163
Tmem	*Tmem131l*	14	16	1.000	*Tmem131l*	13	15	1.000
Eif	*Eif2s3y*	14	15	0.522	*Eif2d*	13	14	0.180
Ptp	*Ptpn9*	13	26	0.889	*Ptpn9*	12	24	0.121
Kif	*Kif23*	12	24	1.000	*Kif23*	10	21	1.000
Ank	*Ank2*	12	35	0.251	*Ank2*	**12**	**33**	**0.047**
Cac	*Cacul1*	11	24	1.000	*Cacul1*	9	18	1.000
Rab	*Rab11a*	10	11	0.355	*Rab11a*	9	10	1.000
**Ppp**	*Ppp1r12*	*10*	*12*	*0.071*	*Ppp1r12a*	8	10	1
Kcn	*Kcnn1*	10	18	1.000	*Kcnn1*	9	16	1.000
Tra	*Tram1*	9	11	1.000	*Traf7*	9	11	1.000
Myo	*Myo9b*	9	19	1.000	*Myo9b*	9	18	1.000
Cam	*Camsap2*	9	24	0.665	*Camk2b*	10	26	1.000
Usp	*Usp37*	9	10	**0.010**	*Usp37*	11	13	0.200

## Data Availability

The RNA-Seq datasets are available in the European Nucleotide Archive (accession no. PRJEB48789).

## Supplementary Materials

The following supporting information can be downloaded at: https://www.mdpi.com/article/10.3390/genes14030599/s1, Table S1. Mapping Stats.xlsx; Table S2a–k. DAS genes tables.xlsx; Table S3a–c. DAS-DEGs genes annotation.xlsx; Table S4a–d. DAS genes protein coding ES events.xlsx; Table S5a–d. DAS merged genes AS2 NMD events.xlsx; Table S6a–d. Top AS2 ES abundant genes.xls; Table S7a–c. Top NMD ES abundant genes.xlsx; Table S8a–c, Splicing Factors analysis.

## Abbreviations

DASs	Differentially alternatively spliced genes
FPKM	Fragments per kilobase of transcript per million mapped reads
GO	Gene ontology
KEGG	Kyoto Encyclopedia of Genes and Genomes Pathway Database
DEGs	Differentially expressed genes
ES	Exon skipping
AS2	Alternative splicing of ES exon with both isoforms coding
NMD	Nonsense-mediated decay

## References

[1] Kudryavtseva N.N., O’Neal P.W. (2007). Straub tail, the deprivation effect and addiction to aggression. Motivation of Health Behavior.

[2] Kudryavtseva N.N., Smagin D.A., Bondar N.P. (2011). Modeling fighting deprivation effect in mouse repeated aggression paradigm. Prog. Neuropsychopharmacol. Biol. Psychiatry.

[3] Kudryavtseva N.N. (2020). Positive fighting experience, addiction-like state, and relapse: Retrospective analysis of experimental studies. Aggress. Violent Behav..

[4] Babenko V., Redina O., Smagin D., Kovalenko I., Galyamina A., Babenko R., Kudryavtseva N. (2021). Dorsal Striatum Transcriptome Profile Profound Shift in Repeated Aggression Mouse Model Converged to Networks of 12 Transcription Factors after Fighting Deprivation. Genes.

[5] Raj B., Blencowe B.J. (2015). Alternative Splicing in the Mammalian Nervous System: Recent Insights into Mecha-nisms and Functional Roles. Neuron.

[6] Smith A.C.W., Jonkman S., Difeliceantonio A.G., O’Connor M.R., Ghoshal S., Romano M.F., Everitt B.J., Kenny P.J. (2021). Opposing roles for striatonigral and striatopallidal neurons in dorsolateral striatum in consoli-dating new instrumental actions. Nat. Commun..

[7] Nishi A., Matamales M., Musante V., Valjent E., Kuroiwa M., Kitahara Y., Rebholz H., Greengard P., Girault J.-A., Nairn A.C. (2017). Glutamate Counteracts Dopamine/PKA Signaling via Dephosphorylation of DARPP-32 Ser-97 and Alteration of Its Cytonuclear Distribution. J. Biol. Chem..

[8] Takahashi A., Chung J.-R., Zhang S., Zhang H., Grossman Y., Aleyasin H., Flanigan M.E., Pfau M.L., Menard C., Dumitriu D. (2017). Establishment of a repeated social defeat stress model in female mice. Sci. Rep..

[9] Yapo C., Nair A.G., Clement L., Castro L.R., Hellgren Kotaleski J., Vincent P. (2017). Detection of phasic do-pamine by D1 and D2 striatal medium spiny neurons. J. Physiol..

[10] Babenko V.N., Galyamina A.G., Rogozin I.B., Smagin D.A., Kudryavtseva N.N. (2020). Dopamine response gene pathways in dorsal striatum MSNs from a gene expression viewpoint: cAMP-mediated gene networks. BMC Neurosci..

[11] Hikida T., Morita M., Kuroiwa M., Macpherson T., Shuto T., Sotogaku N., Niwa M., Sawa A., Nishi A. (2020). Adolescent psychosocial stress enhances sensitization to cocaine exposure in genetically vulnerable mice. Neurosci. Res..

[12] Plattner F., Hayashi K., Hernandez-Cortes A., Benavides D.R., Tassin T.C., Tan C., Day J.P., Fina M.W., Yuen E.Y., Yan Z. (2015). The role of ventral striatal cAMP signaling in stress-induced behaviors. Nat. Neurosci..

[13] Stamm S. (2008). Regulation of alternative splicing by reversible protein phosphorylation. J. Biol. Chem..

[14] Naro C., Sette C. (2013). Phosphorylation-mediated regulation of alternative splicing in cancer. Int. J. Cell. Biol..

[15] Ruta V., Pagliarini V., Sette C. (2021). Coordination of RNA Processing Regulation by Signal Transduction Pathways. Biomolecules.

[16] Xu S.J., Lombroso S.I., Fischer D.K., Carpenter M.D., Marchione D.M., Hamilton P.J., Lim C.J., Neve R.L., Garcia B.A., Wimmer M.E. (2021). Chromatin-mediated alternative splicing regulates cocaine-reward behavior. Neuron.

[17] Lopez Soto E.J., Lipscombe D. (2020). Cell-specific exon methylation and CTCF binding in neurons regulate calcium ion channel splicing and function. Elife.

[18] Alharbi A.B., Schmitz U., Bailey C.G., Rasko J.E.J. (2021). CTCF as a regulator of alternative splicing: New tricks for an old player. Nucleic Acids Res..

[19] Schwartz S., Ast G. (2010). Chromatin density and splicing destiny: On the cross-talk between chromatin structure and splicing. EMBO J..

[20] Mazin P.V., Khaitovich P., Cardoso-Moreira M., Kaessmann H. (2021). Alternative splicing during mammalian organ development. Nat. Genet.

[21] Zheng S. (2016). Alternative splicing and nonsense-mediated mRNA decay enforce neural specific gene expression. Int. J. Dev. Neurosci..

[22] Jaffrey S.R., Wilkinson M.F. (2018). Nonsense-mediated RNA decay in the brain: Emerging modulator of neural development and disease. Nat. Rev. Neurosci..

[23] Zhang Y., Chen K., Sloan S.A., Bennett M.L., Scholze A.R., O’Keeffe S., Phatnani H.P., Guarnieri P., Caneda C., Ruderisch N. (2014). An RNA-Sequencing Transcriptome and Splicing Database of Glia, Neurons, and Vascular Cells of the Cerebral Cortex. J. Neurosci..

[24] Kudryavtseva N.N. (1991). A sensory contact model for the study of aggressive and submissive behavior in male-mice. Aggress. Behav..

[25] Kudryavtseva N.N., Smagin D.A., Kovalenko I.L., Vishnivetskaya G.B. (2014). Repeated positive fighting experience in male inbred mice. Nat. Protoc..

[26] Robinson T.E., Berridge K.C. (2003). Addiction. Annu. Rev. Psychol..

[27] Allen Mouse Brain Atlas. http://mouse.brain-map.org/static/atlas.

[28] Bolger A.M., Lohse M., Usadel B. (2014). Trimmomatic: A flexible trimmer for Illumina sequence data. Bioinformatics.

[29] Dobin A., Gingeras T.R. (2015). Mapping RNA-seq Reads with STAR. Curr. Protoc. Bioinform..

[30] Grau-Bové X., Ruiz-Trillo I., Irimia M. (2018). Origin of exon skipping-rich transcriptomes in animals driven by evolution of gene architecture. Genome Biol..

[31] McGuire A.M., Pearson M.D., Neafsey D.E., Galagan J.E. (2008). Cross-kingdom patterns of alternative splicing and splice recognition. Genome Biol..

[32] Hide W.A., Babenko V.N., van Heusden P.A., Seoighe C., Kelso J.F. (2001). The contribution of exon-skipping events on chromosome 22 to protein coding diversity. Genome Res..

[33] Shen S., Park J.W., Lu Z.X., Lin L., Henry M.D., Wu Y.N., Zhou Q., Xing Y. (2014). rMATS: Robust and flexible detection of differential alternative splicing from replicate RNA-Seq data. Proc. Natl. Acad. Sci. USA.

[34] Robinson P.N., Webber C. (2014). Phenotype ontologies and cross-species analysis for translational research. PLoS Genet..

[35] Naftelberg S., Schor I.E., Ast G., Kornblihtt A.R. (2015). Regulation of alternative splicing through coupling with transcription and chromatin structure. Annu. Rev. Biochem..

[36] Kalmykova S., Kalinina M., Denisov S., Mironov A., Skvortsov D., Guigó R., Pervouchine D. (2021). Conserved long-range base pairings are associated with pre-mRNA processing of human genes. Nat. Commun..

[37] Schärfen L., Neugebauer K.M. (2021). Transcription Regulation Through Nascent RNA Folding. J. Mol. Biol..

[38] Caizzi L., Monteiro-Martins S., Schwalb B., Lysakovskaia K., Schmitzova J., Sawicka A., Chen Y., Lidschreiber M., Cramer P. (2021). Efficient RNA polymerase II pause release requires U2 snRNP function. Mol. Cell.

[39] Gordon J.M., Phizicky D.V., Neugebauer K.M. (2021). Nuclear mechanisms of gene expression control: Pre-mRNA splicing as a life or death decision. Curr. Opin. Genet. Dev..

[40] La Manno G., Soldatov R., Zeisel A., Braun E., Hochgerner H., Petukhov V., Lidschreiber K., Kastriti M.E., Lön-nerberg P., Furlan A. (2018). RNA velocity of single cells. Nature.

[41] Huang L., Lou C.H., Chan W., Shum E.Y., Shao A., Stone E., Karam R., Song H.W., Wilkinson M.F. (2011). RNA homeo-stasis governed by cell type-specific and branched feedback loops acting on NMD. Mol. Cell.

[42] Dar R.D., Razooky B.S., Singh A., Trimeloni T.V., McCollum J.M., Cox C.D., Simpson M.L., Weinberger L.S. (2012). Tran-scriptional burst frequency and burst size are equally modulated across the human genome. Proc. Natl. Acad. Sci. USA.

[43] Nicolas D., Zoller B., Suter D.M., Naef F. (2018). Modulation of transcriptional burst frequency by histone acetylation. Proc. Natl. Acad. Sci. USA.

[44] Hermey G., Blüthgen N., Kuhl D. (2017). Neuronal activity-regulated alternative mRNA splicing. Int. J. Biochem. Cell Biol..

[45] Chen L.F., Lin Y.T., Gallegos D.A., Hazlett M.F., Gómez-Schiavon M., Yang M.G., Kalmeta B., Zhou A.S., Holtzman L., Gersbach C.A. (2019). Enhancer Histone Acetylation Modulates Transcriptional Bursting Dynamics of Neuronal Activity-Inducible Genes. Cell Rep..

[46] Vivinetto A.L., Kim I.D., Goldberg D.C., Fones L., Brown E., Tarabykin V.S., Hill C.E., Cho S., Cave J.W. (2020). Zeb2 Is a Regulator of Astrogliosis and Functional Recovery after CNS Injury. Cell Rep..

[47] Yan Q., Weyn-Vanhentenryck S.M., Wu J., Sloan S.A., Zhang Y., Chen K., Wu J.Q., Barres B.A., Zhang C. (2015). Systematic discovery of regulated and conserved alternative exons in the mammalian brain reveals NMD modulating chromatin regulators. Proc. Natl. Acad. Sci. USA.

[48] Stucchi R., Plucińska G., Hummel J.J.A., Zahavi E.E., Guerra San Juan I., Klykov O., Scheltema R.A., Altelaar A.F.M., Hoogenraad C.C. (2018). Regulation of KIF1A-Driven Dense Core Vesicle Transport: Ca2+/CaM Controls DCV Binding and Liprin-α/TANC2 Recruits DCVs to Postsynaptic Sites. Cell Rep..

[49] Ashraf S., Gee H.Y., Woerner S., Xie L.X., Vega-Warner V., Lovric S., Fang H., Song X., Cattran D.C., Avila-Casado C. (2013). ADCK4 mutations promote steroid-resistant nephrotic syndrome through CoQ10 biosynthesis disruption. J. Clin. Investig..

[50] Petruzzella V., Panelli D., Torraco A., Stella A., Papa S. (2005). Mutations in the NDUFS4 gene of mitochondrial complex I alter stability of the splice variants. FEBS Lett..

[51] Panelli D., Lorusso F., Papa F., Sardanell A., Papa S. (2013). Alternative splicing and nonsense mediated decay in mitochondrial complex-I biogenesis and its implication in human diseases. J. Bioanal. Biomed..

[52] Mironov A., Denisov S., Gress A., Kalinina O.V., Pervouchine D.D. (2021). An extended catalogue of tandem alternative splice sites in human tissue transcriptomes. PLoS Comput. Biol..

[53] Jangi M., Sharp P.A. (2014). Building robust transcriptomes with master splicing factors. Cell.

[54] Jangi M., Boutz P.L., Paul P., Sharp P.A. (2014). Rbfox2 controls autoregulation in RNA-binding protein networks. Genes Dev..

[55] Pervouchine D., Popov Y., Berry A., Borsari B., Frankish A., Guigó R. (2019). Integrative transcriptomic analysis suggests new autoregulatory splicing events coupled with nonsense-mediated mRNA decay. Nucleic Acids Res..

[56] Vuong C.K., Black D.L., Zheng S. (2016). The neurogenetics of alternative splicing. Nat. Rev. Neurosci..

[57] Vuong J.K., Lin C.H., Zhang M., Chen L., Black D.L., Zheng S. (2016). PTBP1 and PTBP2 Serve Both Specific and Redundant Functions in Neuronal Pre-mRNA Splicing. Cell Rep..

[58] Makeyev E.V., Maniatis T. (2008). Multilevel regulation of gene expression by microRNAs. Science.

[59] Keppetipola N.M., Yeom K.H., Hernandez A.L., Bui T., Sharma S., Black D.L. (2016). Multiple determinants of splicing repression activity in the polypyrimidine tract binding proteins, PTBP1 and PTBP2. RNA.

[60] Lee J.A., Xing Y., Nguyen D., Xie J., Lee C.J., Black D.L. (2007). Depolarization and CaM kinase IV modulate NMDA receptor splicing through two essential RNA elements. PLoS Biol..

[61] Porter R.S., Jaamour F., Iwase S. (2018). Neuron-specific alternative splicing of transcriptional machineries: Implications for neurodevelopmental disorders. Mol. Cell Neurosci..

[62] Pandey S.C., Ugale R., Zhang H., Tang L., Prakash A. (2008). Brain chromatin remodeling: A novel mechanism of alcoholism. J. Neurosci..

[63] Rahhal R., Seto E. (2019). Emerging roles of histone modifications and HDACs in RNA splicing. Nucleic Acids Res..

[64] Furlanis E., Traunmüller L., Fucile G., Scheiffele P. (2019). Landscape of ribosome-engaged transcript isoforms reveals extensive neuronal-cell-class-specific alternative splicing programs. Nat. Neurosci..

[65] Gañez-Zapater A., Mackowiak S.D., Guo Y., Tarbier M., Jordán-Pla A., Friedländer M.R., Visa N., Östlund Far-rants A.K. (2022). The SWI/SNF subunit BRG1 affects alternative splicing by changing RNA binding factor interac-tions with nascent RNA. Mol. Genet. Genom..

[66] Batsché E., Yaniv M., Muchardt C. (2006). The human SWI/SNF subunit Brm is a regulator of alternative splicing. Nat. Struct Mol. Biol..

[67] Kazantseva A., Sepp M., Kazantseva J., Sadam H., Pruunsild P., Timmusk T., Neuman T., Palm K. (2009). N-terminally truncated BAF57 isoforms contribute to the diversity of SWI/SNF complexes in neurons. J. Neurochem..

[68] Gueroussov S., Gonatopoulos-Pournatzis T., Irimia M., Raj B., Lin Z.Y., Gingras A.C., Blencowe B.J. (2015). An alternative splicing event amplifies evolutionary differences between vertebrates. Science.

[69] Su C.H., Tarn W.Y. (2018). Alternative Splicing in Neurogenesis and Brain Development. Front. Mol. Biosci..

[70] Ho W.Y., Agrawal I., Tyan S.H., Sanford E., Chang W.T., Lim K., Ong J., Tan B.S.Y., Moe A.A.K., Yu R. (2021). Dysfunction in nonsense-mediated decay, protein homeo-stasis, mitochondrial function, and brain connectivity in ALS-FUS mice with cognitive deficits. Acta Neuro-Pathol. Commun..

[71] Sikora M., Tokarski K., Bobula B., Zajdel J., Jastrzębska K., Cieślak P.E., Zygmunt M., Sowa J., Smutek M., Kamińska K. (2016). NMDA Receptors on Dopaminoceptive Neurons Are Essential for Drug-Induced Conditioned Place Preference. eNeuro.

[72] Nakanishi N., Axel R., Shneider N.A. (1992). Alternative splicing generates functionally distinct N-methyl-D-aspartate receptors. Proc. Natl. Acad. Sci. USA.

[73] Ehlers M.D., Tingley W.G., Huganir R.L. (1995). Regulated subcellular distribution of the NR1 subunit of the NMDA receptor. Science.

[74] Yap K., Xiao Y., Friedman B.A., Friedman B.A., Je H.S., Makeyev E.V. (2016). Polarizing the neuron through sustained co-expression of alternatively spliced isoforms. Cell Rep..

[75] Bradley J., Carter S.R., Rao V.R., Wang J., Finkbeiner S. (2006). Splice variants of the NR1 subunit differentially induce NMDA receptor-dependent gene expression. J. Neurosci..

[76] Patthy L. (2019). Exon skipping-rich transcriptomes of animals reflect the significance of exon-shuffling in metazoan proteome evolution. Biol. Direct.

